# Links between Aggressive Sexual Fantasies and Sexual Coercion: A Replication and Extension of a Multifactorial Model

**DOI:** 10.1007/s10508-023-02782-5

**Published:** 2024-01-17

**Authors:** Joseph Bernhard Birke, Patrick Jern, Ada Johansson, Rebecca Bondü

**Affiliations:** 1 Department of Psychology, Psychologische Hochschule Berlin, Am Köllnischen Park 2, 10179 Berlin, Germany; 2 Department of Psychology and Logopedics, Abo Akademi University, Turku, Finland

**Keywords:** Aggressive sexual fantasies, Sexual coercion, Multifactor model, Structural equation model

## Abstract

Current research indicates that aggressive sexual fantasies (ASF) are related to sexual aggression, above and beyond other risk factors for this behavior. There have, however, rarely been explicitly considered in multifactor models aiming to explain sexual aggression. One exception is the multifactorial Revised Confluence Model of Sexual Aggression that was replicated in two samples of male individuals who were convicted of sexual offenses and a small sample of men from the general population and evidenced a high relevance of ASF, respectively. There were, however, no further attempts to replicate the model in larger samples from the general population. We, therefore, used a subsample from the Finnish Genetics of Sexuality and Aggression project including 3269 men (age: *M* = 26.17 years, *SD* = 4.76) to do so. Cross-sectional latent structural equation models corroborated previous research and the assumption that ASF are a central component in multifactor models that aim to explain sexual aggression: ASF and antisocial behavior/aggression were equally important associates of sexual coercion when also considering adverse childhood experiences, hypersexuality, and callous-unemotional traits. Additionally, ASF mediated the links between hypersexuality, callous-unemotional traits, as well as childhood sexual abuse and sexual coercion. These links held stable when entering further risk factors, that is, distorted perceptions, rape-supportive attitudes, and violent pornography consumption into the model. Contrasting assumptions, alcohol consumption and antisocial behavior/aggression did not interact. These results illustrate the potential importance of ASF for sexual aggression. They indicate that ASF require consideration by research on sexual aggression as well as in the treatment and risk assessment of sexual perpetrators.

**Supplementary Information:**

The online version contains supplementary material available at 10.1007/s10508-023-02782-5.

## Introduction

Research has shown that fantasizing about physically or psychologically harming another person to reach sexual arousal (i.e., aggressive sexual fantasies; ASF) is associated with sexual aggression beyond other risk factors for this behavior in both samples of male individuals convicted of sexual offenses (Knight & Sims-Knight, 2004, 2006; Prentky et al., 1989; Woodworth et al., 2013) and community samples (Birke & Bondü, 2022; Bondü & Birke, 2021; Knight & Sims-Knight, 2006). Thus, ASF can be considered a close associate of sexual coercion. However, recent research on the incremental validity of ASF for this behavior has not considered all relevant control variables and potential predictors of ASF itself. It has often used similar measures of ASF, making it questionable whether the associated findings can be replicated with other measures of the construct. Finally, recent research on ASF often used potentially consensual, sadistic, instead of clearly non-consensual, coercive behavior as the outcome measure. Thus, the present study aimed to close these gaps in research.

Although ASF have often been described as potential risk factors for sexual aggression (Ronis et al., 2019), they were rarely considered in theoretical models that aim to explain this behavior. One exception is the multifactor model by Knight and Sims-Knight (2004, 2006) which adapted the confluence model (Malamuth, 1998; Malamuth et al., 2021) by including ASF besides other important risk factors for sexual coercion, such as callous-unemotional traits, antisocial behavior/aggression, and hypersexuality. The model was empirically supported in samples of male individuals who have sexually offended and a sample from the general public (Knight & Sims-Knight, 2004, 2006). The population-based sample, however, was rather small. Furthermore, not all relevant paths were tested in the study (Knight & Sims-Knight, 2006), and some relevant variables were not considered in the statistical models. The present study, therefore, aimed to replicate the original multifactor model via a secondary data analysis of a large male sample from the general public using cross-sectional data, while considering additional potentially relevant risk factors and pathways. This allowed for highlighting the importance of ASF as an associate of sexual coercion relative to a broad range of other relevant variables and for determining whether previous findings in samples of male individuals convicted of a sexual offense can be generalized to community samples. In other words, we explored whether a “unified theory of sexual aggression” can be applied to sexual perpetrators with and without criminal record (Knight & Sims-Knight, 2006; Malamuth, 2006). This knowledge may aid to derive effective intervention and prevention strategies to avoid sexual offending. In addition, re-investigating a complex model by a team of independent researchers using an already existing data set addresses the demand for replication in psychological research.

### Sexual Aggression

Sexual aggression is defined as any sexual behavior with someone who does not or cannot consent to engage in this behavior (Basile et al., 2014). It is a widespread phenomenon (Garcia-Moreno et al., 2005; Jewkes & Dartnall, 2017): An estimated 6% of all women globally have reportedly experienced non-partner sexual violence after the age of 15, and 26% of ever-partnered women experienced physical and/or sexual intimate-partner violence at least once (World Health Organization, 2021). Accordingly, sexual offending is also frequent: For male samples, research yielded evidence for prevalence rates between 10 and 35%, with more severe forms of sexual aggression showing lower prevalence rates (Abbey & McAuslan, 2004; Krahé et al., 2021; Schuster et al., 2016; White & Smith, 2004). Of note, large proportions of sexual offenses do not become known to law enforcement authorities, making sexual aggression a problem not only in forensic contexts, but also in the general population.

Experiencing sexual aggression often impairs the victim’s physical or psychological functioning and is correlated with anxiety, depression, substance abuse, suicidal behavior, and post-traumatic stress disorder (Jina & Thomas, 2013). Thus, it is pivotal to identify factors that are associated with sexual aggression to prevent it in the first place and to improve interventions to diminish recidivism (Mann et al., 2010). These risk factors include general, underlying, or motivating factors (e.g., hypersexuality, antisocial traits and behavior, aggression-related sexual interests) as well as specific, situational, or facilitating factors (e.g., rape myth acceptance, hostile attitudes toward women, alcohol consumption) (Seto, 2019). In addition, recent research has pointed to ASF as a further discrete factor (Bartels et al., 2020; Birke & Bondü, 2022; Bondü & Birke, 2021) that may bridge these two groups of factors.

### Aggression-Related Sexual Fantasies

Based on the definition of sexual fantasies as “any mental imagery that is sexually arousing or erotic to the individual” (Leitenberg & Henning, 1995, p. 470), in the present study, ASF are defined as sexually arousing cognitions ranging from fleeting thoughts to active fantasizing about the infliction of physical or mental harm onto another person, irrespective of the consent of the other person (Bartels & Beech, 2016; Bondü & Birke, 2020, 2021). These fantasies can comprise cognitions of both, consensual harmful and clearly non-consensual sexually aggressive acts, each of which may be aligned with different paraphilia (Harris et al., 2012; Martin et al., 2016; Seto et al., 2012).

ASF about non-consensual, coercive, or sexually violent acts have been reported by both, men convicted of sexual offenses and from community samples. Between 50 and 80% of men convicted of sexual offenses reported ASF about sexual assault and violence (Leitenberg & Henning, 1995) and between 8 and 38% of participants from community samples reported fantasies about coercing someone into sex or raping someone (Bártová et al., 2021; Bondü & Birke, 2021; Hsu et al., 1994; Joyal et al., 2015; Person et al., 1989). In men convicted of sexual offenses, the sexual-fantasy contents corresponded to the features of their offenses (Woodworth et al., 2013), ASF were described as “cognitive try-outs” (Prentky et al., 1989), considered as a mean for mood regulation (McKibben et al., 1994; Proulx et al., 1996), and predicted sexual coercion with beta weights of 0.44-0.66 (Knight & Sims-Knight, 2004, 2006). In the general population, a composite score reflecting a broad spectrum of ASF predicted various forms of harmful sexual behavior with beta weights of 0.28-0.56 cross-sectional (Birke & Bondü, 2022; Bondü, 2023; Bondü & Birke, 2020, 2021; Knight & Sims-Knight, 2006).

The adverse effects of ASF may be explained within the framework of script theory (Huesmann, 1998) that assumes that aggressive behavior is organized according to scripts that guide responses to specific stimuli and situations. This assumption has also been transferred to sexual behavior and sexual aggression (Ward & Hudson, 2000). The model suggests that cognitive re-iterations of (sexually) aggressive thoughts—such as ASF— strengthen and elaborate cognitive scripts for (sexual) aggression and make these scripts more easily accessible and activated as well as more frequently relevant in a larger number of situations. Additionally, ASF can trigger and/or increase sexual arousal that may impair sexual inhibition and decision making and, thereby, increase the likelihood of sexual aggression (Imhoff & Schmidt, 2014; Smid & Wever, 2019). However, not only ASF frequency, but also their evaluation may be related to sexual aggression (Bondü, 2023), although findings have been ambiguous: One study showed positive correlations between ASF and positive attitudes toward one’s own sexual fantasies in general, but no correlation with negative attitudes, whereas regression analyses showed the reverse pattern (Bondü & Birke, 2021). Another study found positive correlations between evaluations of dominance fantasies as pleasant and self-reported sexual aggression (Renaud & Byers, 2005). From a theoretical point of view, positive attitudes toward one’s own ASF should be expected to increase their frequency and elaboration which should in turn increase the urge to put them into action (Bartels & Beech, 2016; Bondü, 2023).

Taken together, theoretical assumptions and empirical evidence show that ASF may be an important correlate of sexual aggression in both men convicted of sexual offenses and community samples. However, more research is needed to highlight the relative importance of ASF as a correlate of sexual aggression, to disentangle the associations between ASF and other risk factors, and to identify potential antecedents of ASF. Indeed, ASF have been found to be associated also with other risk factors for sexual aggression. They were positively related to hypersexuality and sex drive (Knight & Sims-Knight, 2004, 2006), psychopathy, aggressive and antisocial behavior, and hostility (Baughman et al., 2014; Bondü & Birke, 2021; Knight & Sims-Knight, 2004, 2006; Williams et al., 2009; Woodworth et al., 2013), violent pornography consumption (Bondü & Birke, 2021), as well as rape-supportive attitudes, such as rape myth acceptance, hostile beliefs about women, and rape proclivity (Bartels & Gannon, 2009; Bartels et al., 2020; Bondü & Birke, 2021; Dean & Malamuth, 1997). Self-reported ASF showed small to high correlations with self-reports as well as direct and indirect assessments of sexual interest in pain infliction, humiliation, and coercion (*r* = 0.09–0.80) (Bártová et al., 2021; Birke & Bondü, 2022; Larue et al., 2014) and zero to moderate correlations with directly and indirectly measured sexual preferences for violent and sexually violent stimuli (*r* = 0.00–0.39) (Birke & Bondü, 2022; Larue et al., 2014).

Close associations between ASF and sexual interests as well as violent pornography consumption indicated that ASF may be considered as an indicator of accordant sexual interests. Indeed, most theories on sexual aggression have not considered ASF as a relevant factor (Dean & Malamuth, 1997) or merely as an indicator of accordant sexual interests (Seto, 2019). This notion, however, is contrasted by recent research that showed ASF to predict sexual aggression better than the sexual preference for violent and sexually violent stimuli, and better than violent pornography consumption (Birke & Bondü, 2022; Bondü & Birke, 2021), indicating the incremental validity of ASF. Consequently, conceptualizing ASF only as an indicator of sexual interests may underestimate their relevance for sexual aggression.

**Fig. 1:**
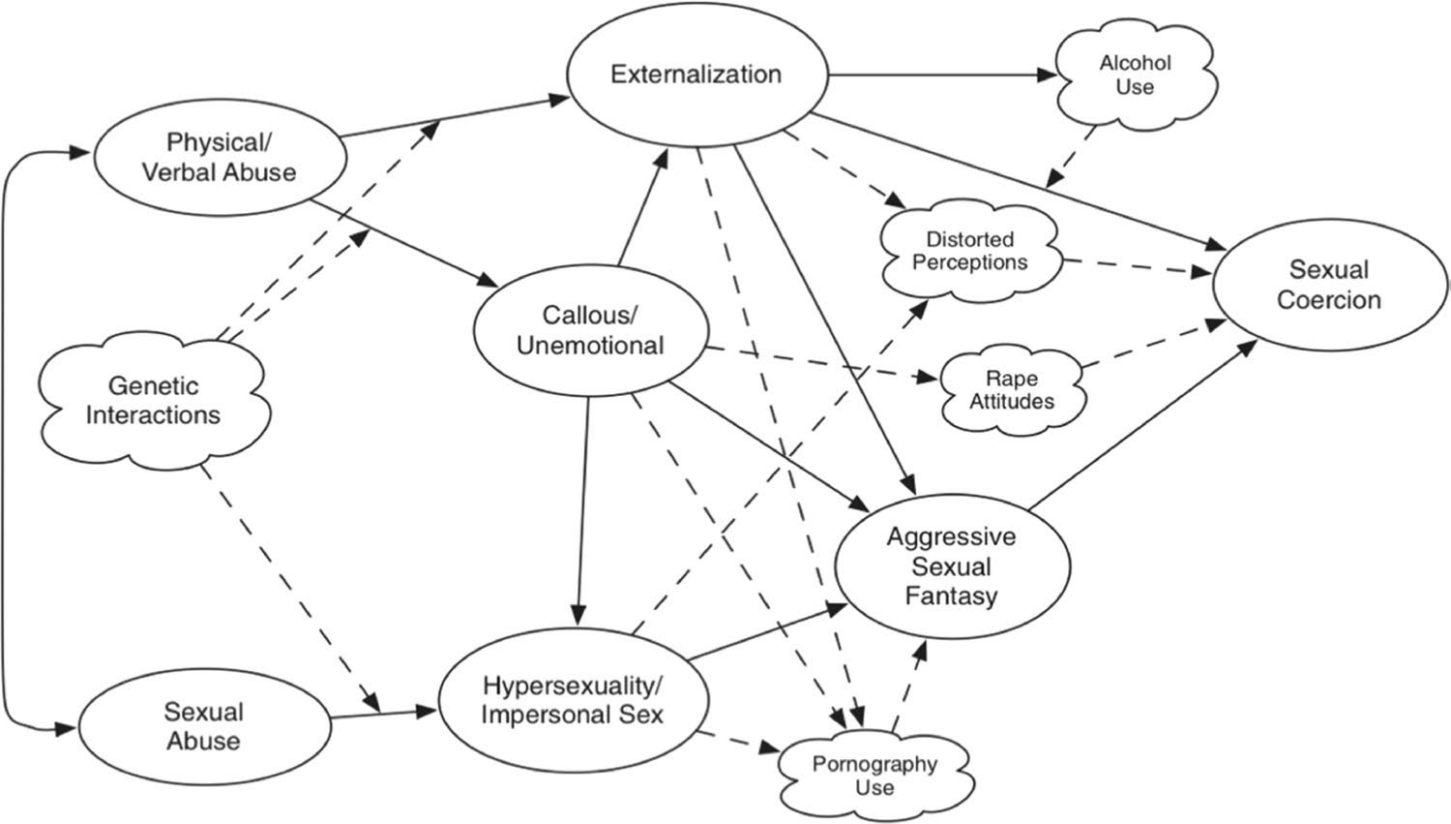
Multifactor model by Knight and Sims-Knight (2011) Including Suggested Extensions. *Note.* Figure 6.2 from Knight and Sims-Knight (2011, p. 131). Variables in ovals have been included in the original model (Knight & Sims-Knight, 2004, 2006). Variables in clouds are suggestions for an extension of the model (Knight & Sims-Knight, 2011)

Positive associations between ASF and positive attitudes toward one’s own sexual fantasies and rape-supporting attitudes (Bartels et al., 2020; Bondü & Birke, 2021; Dean & Malamuth, 1997), as well as their conceptualization as cognitive try-outs (Prentky et al., 1989), indicate that ASF may not only motivate toward sexual aggression similar to accordant sexual interests (Seto, 2019). They may also facilitate such behavior by cognitively practicing and thereby lowering the thresholds for such behavior. Nonetheless, most theories on sexual aggression have not considered ASF at all or merely as an indicator of accordant sexual interests (Seto, 2019). One model that has considered ASF as a central factor in explaining sexual aggression is the multifactorial model for the explanation of sexual coercion by Knight and Sims-Knight (2004, 2006). This model, therefore, is a promising starting point for research on the position of ASF in the multifactorial framework of sexual aggression (Fig. 1).

### A Multifactorial Model for Sexual Aggression

The aforementioned multifactor model elaborated on the confluence model of sexual aggression that aims to explain sexual aggression in community samples. The confluence model suggests two major pathways toward sexual aggression. On the first pathway, own experiences of maltreatment and abuse during childhood cause delinquent behavior. The effects of this behavior on sexual aggression are then mediated by impersonal sex (i.e., detachment of sexuality and emotions). On the second pathway, the effects of psychopathy on sexual aggression are mediated by hostile masculinity (i.e., hostile attitudes toward women or distorted beliefs, such as that women want to be raped). In addition, impersonal sex and hostile masculinity interacted in predicting sexual aggression (Malamuth et al., 1991, 2021).

Knight and colleagues adapted this model to samples of male individuals convicted of sexual offenses and outlined three instead of two potential pathways to sexual aggression by adding callous-unemotional traits as a third core risk factor. In addition, instead of hostile masculinity and impersonal sex, they included ASF as the central mediating variable of all three pathways into the model. Accordingly, the model has sometimes been referred to as the revised confluence model of sexual aggression. On the first path, physical and verbal abuse experiences during childhood (indicated by self-reported verbal abuse or neglect and the severity and frequency of physical abuse) directly as well as indirectly through callous-unemotional traits (indicated by negative masculinity, superficial charm as a facet of psychopathy, and impulsivity; thus, this factor integrates aspects of hostile masculinity from the original Confluence model into a broader factor of antisocial traits) foster antisocial behavior/aggression (indicated by adolescent problem behavior, bullying as one important recurrent form of aggression, and alcohol consumption). Antisocial behavior/aggression, in turn, is assumed to directly and indirectly via ASF (indicated by aggressive fantasies about women and sadistic behavior) predict sexual coercion (indicated by a range of coercive acts). The second path assumes physical and verbal abuse experiences during childhood to promote callous-unemotional traits that indirectly increase the risk for sexual coercion by promoting ASF. The third path assumes that early experiences of sexual abuse (indicated by the form of perpetration, amount of force, number of perpetrators, and age at abuse) and callous-unemotional traits promote hypersexuality (indicated by sexual compulsivity, high sex drive, and sexual preoccupation, thereby integrating aspects of impersonal sex form the original confluence model into this factors, but treating it as an independent rather than a mediating factor) that indirectly increases the risk for sexual coercion by promoting ASF (Fig. 1).

The model was first tested in 275 men who have sexually offended and then replicated in 218 male adolescents convicted of a sexual offense and 168 men from a community sample using structural equation models with satisfying to good fits with the data, respectively. The models explained 44–88% of the variance in ASF and 33–65% of the variance in sexual coercion. ASF were the strongest predictor of sexual coercion with beta-weights between 0.44 and 0.66 cross-sectional (Knight & Sims-Knight, 2004, 2006), followed by antisocial behavior/aggression. These results point to an important role of ASF in explaining variance in sexual coercion. They also suggest that ASF may be influenced by hypersexuality, antisocial behavior/aggression, and callous-unemotional traits, although there were no associations between ASF and antisocial behavior/aggression in the sample of male adolescents convicted of a sexual offense and no associations with callous-unemotional traits in the normal-population sample. The expected association between sexual abuse and hypersexuality could not be replicated, but post hoc analyses suggested direct associations between sexual abuse and sexual coercion in the sample of adolescent males convicted of a sexual offense (Knight & Sims-Knight, 2004, 2006). Given these variations in findings, that the model was derived from a sample of men who have sexually offended, and that it was mostly tested in small samples, it seems important to replicate it in a larger, population-based sample to examine whether all pathways should be upheld. Additionally, not all potential pathways were estimated by the previous studies. Therefore, it is not yet clear whether there may be further direct or indirect effects between the variables that have not yet been detected.

### Extending the Multifactorial Model of Sexual Aggression

Finally, there are additional well-established risk factors for sexual aggression that the model has not yet considered. Therefore, Knight and Sims-Knight (2011) suggested an extension of their original model that also included distorted perceptions, rape-supportive attitudes, pornography consumption, and alcohol consumption. These variables are not only relevant in forensic research, but are also considered as important risk factors for sexual aggression in the normal population by social psychological research (Abbey et al., 2011; Malamuth et al., 2021) and may, therefore, be particularly important when trying to replicate the model in a community sample.

Distorted perceptions include persistent beliefs that may promote sexual aggression, such as “women are sex objects” or rape myths, such as “women want to be raped” (Beech et al., 2005). These beliefs were found in and showed associations with recidivism in men who sexually offended (Helmus et al., 2013). In the general population, rape myth acceptance was positively correlated with sexual aggression (Gerger et al., 2007; Lonsway & Fitzgerald, 1994). In the extended multifactorial model, distorted perceptions were considered as an additional mediator of antisocial behavior/aggression and hypersexuality. Impulsive tendencies associated with antisocial behavior/aggression and sexually preoccupied cognitions may increase the tendency toward sexual aggression and lead to the development of distorted assumptions about women’s sexual wishes that are needed to justify sexual coercive behavior.

Rape-supportive attitudes typically comprise rape myth acceptance, adversarial beliefs about women, or the acceptance of interpersonal violence, all of which have been positively correlated with sexual aggression (Burt, 1980; Lonsway & Fitzgerald, 1995; Malamuth, 1989a, 1989b). Furthermore, rape-supportive attitudes include positive evaluations of rape itself, which are associated with a higher self-reported likelihood to rape in case of impunity, that is, rape proclivity (Hermann et al., 2018; Nunes et al., 2018). Rape proclivity, therefore, may be considered an indicator of positive evaluations of rape. In the extended multifactorial model, rape-supportive attitudes were considered as an additional mediator of callous-unemotional traits next to ASF. Such positive evaluations may be enabled due to the empathic deficits associated with high expressions of callous-emotional traits, which in turn disinhibit one’s own sexual coercive behavior.

General pornography consumption has only inconsistently been related to sexual aggression (Mellor & Duff, 2019), but the consumption of pornography with violent contents consistently showed positive associations (Bondü & Birke, 2021; Malamuth, 1981; Malamuth & Huppin, 2005; Malamuth et al., 2021). In the extended multifactorial model, it was considered as a mediator between hypersexuality and ASF. Hypersexual tendencies may increase the need for more and more extreme pornographic contents, including violent contents, which in turn may stimulate or promote fantasies about sexual aggression.

Finally, alcohol consumption is a well-established situational risk factor for sexual aggression that strengthens associations between general and sexual aggression (Abbey et al., 2011, 2014). Thus, in the extended multifactorial model, alcohol consumption was considered as a moderator of antisocial behavior/aggression on sexual coercion (Fig. 1).

These extensions connect forensic research in male individuals convicted of sexual offenses focusing on general antisociality and deviant sexuality as well as social psychological research in community samples with a stronger focus on attitudes and beliefs that ease sexual coercion. Testing this comprehensive, “unified model of sexual aggression,” may, therefore, contribute to further develop a general model of sexual aggression and add to the understanding of the relevance of ASF.

### The Present Study

The present study aimed to examine the relative importance and incremental validity of ASF in cross-sectionally explaining variance in sexual coercion and to replicate the original multifactorial model of sexual aggression in a large sample from the general population, and to examine whether the model will improve when other pertinent risk factors for sexual aggression, that is, distorted beliefs, rape-supportive attitudes, violent pornography consumption, and alcohol consumption are entered into the model. To this end, we used cross-sectional data of 3269 men from the Genetics of Sexuality and Aggression (GSA) project (Johansson et al., 2013). In doing so, we add to the existing research by (1) gathering evidence about ASF and their potential associations with other important risk factors for sexual aggression in the statistical prediction of sexual coercion, (2) conceptually replicating a multifactor model from forensic research in a large community sample, and (3) extending the model by important risk factors that are also relevant in social psychological research, thereby taking an essential step toward the idea of a “unified theory of sexual aggression” (Knight & Sims-Knight, 2006; Malamuth, 2006) and providing further insights into the role of ASF for sexual aggression. We expected to replicate the multifactorial model of sexual aggression as shown in Fig. 1 (H1). We expected the inclusion of violent pornography consumption, rape-supportive attitudes, and distorted perceptions as mediators (H2a), and alcohol consumption (H2b) as a moderator into the model to improve the explanation of variance in sexual coercion. We explored models with all possible pathways allowed and estimated to identify potential further relevant pathways, respectively.

## Method

### Participants and Procedure

The present subsample is part of the GSA-project launched at the Abo Akademi University in Turku, Finland. Data were collected in 2006. Participants were recruited via the Central Population Registry of Finland. The study was aimed at all Finnish-speaking twin pairs and their siblings residing in Finland born between July 22, 1973, and March 1, 1988. Potential participants received a letter of inquiry. Participants were required to be at least 18 years old and could answer the survey online or in paper. They could take part in a raffle for four travel vouchers between 500 and 1500€. The ethical board of the university approved the proceedings (Johansson et al., 2013).

The present subsample included male twins and their male siblings who self-reported on their sexual functioning, sexual behavior, and aggressive behavior. It included *N* = 3269 men with a mean age of 26.18 years (SD = 4.76, range 18–48) from 2513 families; 1862 individuals (57%) were the only members of their family in the sample, 1114 individuals (34%) had one sibling in the sample, 255 individuals (8%) had two siblings in the sample, and 38 individuals (1%) had three or more siblings in the sample. 90% of the sample described themselves as exclusively heterosexual.

### Measures

#### Sexual Coercion

We measured sexual coercive behavior via the item “I had or tried to have sex with somebody against their will” from the Self-Report-Psychopathy Scale (Neumann et al., 2012). Response options ranged from 1 (*completely disagree*) to 5 (*completely agree*). In addition, participants were asked whether they had ever had sexual contacts with a person that did not want them to by using six coercive strategies and if so, how intense the potentially resulting coercive act was (e.g., “Have you ever engaged in a sexual interaction with somebody, even if this person did not want to, because you threatened to use physical force?”). If participants reported to have used any of the six coercive strategies, they were subsequently asked to specify, whether this strategy had indeed resulted in 1) kissing and touching the other person and/or 2) oral, vaginal, or anal intercourse, respectively. Both questions had to be answered with 0-*no* or 1-*yes*. If participants reported not to have used any of these strategies, both questions were set to 0. We then calculated a mean score out of the 12 responses that reflected both the range of the different coercive strategies that were used by a person and the intensity of the resulting coercive acts. Both sexual-coercion measures served as indicators to model a latent sexual coercion factor, thereby reducing measurement error.

#### Aggressive Sexual Fantasies

We measured ASF via two items: “How often have you imagined coercing someone into oral, vaginal, or anal sex?” (response options: 0 [*never*] to 2 [*often*]) and “How repulsive or tempting do you find the idea of coercing someone into oral, vagina, or anal sex?” (response options: 1 [*very repulsive*] to 4 [*very tempting*]). Due to the diverging response options, the two items were *z*-standardized and aggregated to a mean for the manifest analyses. In the latent analyses, first, we used both items as indicators of a latent ASF factor that assesses the frequency and evaluation of ASF, and second, both items separately as manifest variables.

#### Callous-Unemotional Traits

We measured callous-unemotional traits via 9 items from the Self-Report-Psychopathy Scale (Neumann et al., 2012; e.g., “I almost never feel guilt for things I have done”) that capture a lack of adequate or moral affective responses. Response options ranged from 1 (*disagree completely*) to 5 (*agree completely*). We computed a mean score following the factor solution reported by Hollerbach et al. (2018) that was partly developed on the basis of the present sample.

#### Antisocial Behavior/Aggression

Antisocial behavior/aggression was measured via 10 items from the SRP-III-A (Neumann et al., 2012; e.g., “Rules are made for breaking”), that capture deviant behavior and an unstable lifestyle. Response options ranged from 1 (*disagree completely*) to 5 (*agree completely*). We computed a mean score following the factor solution reported by Hollerbach et al. (2018) that was partly developed on the basis of the present sample. Additionally, we measured aggressive behavior via 14 items from the Buss and Perry (1992) Aggression Questionnaire (e.g., “If somebody hits me, I hit back”). Response options ranged from 1 (*not typical at all*) to 5 (*very typical*). We computed a mean score. The antisocial behavior and aggression mean scores were used as indicators for the latent factor antisocial behavior/aggression (American Psychiatric Association, 2013; Knight & Sims-Knight, 2011).

#### Hypersexuality

We measured sexual desire with the 6-item sexual desire subscale from the Sexual Desire Inventory (Spector et al., 1996; e.g., “When you have sexual thoughts, how strong is your desire to engage in sexual behavior with a partner?”). Response options ranged from 0 (*no desire*) to 8 (*strong desire*). We measured sex drive with the 12-item sex drive subscale from the Derogatis Sexual Functioning Inventory (DSFI; Derogatis & Melisaratos, 1979). Participants indicated how often they experience different sexual acts in reality and how often they would ideally like to experience them (e.g., masturbation, oral sex, vaginal sex). Response options ranged from 0 (*not at all*) to 8 (> 4* times/day*). We computed mean scores, respectively. Both scores were used as indicators for the latent factor that represents subclinical hypersexuality.

#### Physical, Emotional, and Sexual Abuse

Childhood physical, verbal, and sexual abuse were measured via the respective 5-item subscales from the Childhood Trauma Questionnaire (Bernstein et al., 2003; e.g., physical: “People in my family hit me so hard it left me with bruises or marks”, emotional: “I thought that my parents wished I had never been born”, sexual: “Someone tried to make me do sexual things or watch sexual things”). Response options ranged from 1 (*not at all*) to 5 (*very often*). We computed mean scores for each subscale.

#### Distorted Perceptions

We measured distorted perceptions with one item that captures assumptions about women’s reception of sexual coercion: “How many percent of the female population do you think find coercing someone to oral, vaginal or anal sex sexually arousing?”. Response options ranged from 0 (0%) to 10 (100%).

#### Violent Pornography Consumption

Violent pornography consumption was indicated by one dichotomous item “Have you ever watched porn with elements of coercion, subjugation, or violence?” (0 = *no*/1 = *yes*).

#### Rape-Supportive Attitudes

Rape-supporting attitudes were indicated by two items that assessed the subjective likelihood to rape someone in the case of impunity for oneself and for one’s best friend, respectively. Response options ranged from 1 (*very unlikely*) to 7 (*very likely*). We computed a mean score.

#### Alcohol Consumption

Alcohol consumption was assessed with the 10-item Alcohol Use Disorders Identification Test (Babor et al., 2001). Response options for items assessing the frequency of drinking behavior (e.g., “How many drinks containing alcohol do you have on a typical day when you are drinking?”) ranged from 0 (*never*) to 4 (*daily or almost daily*). Response options for items assessing the consequences of one’s drinking behavior (e.g., “Has a relative, friend, doctor, or other health care worker been concerned about your drinking or suggested you cut down?”) were 0 (*no*), 1 (*yes, but not during the last year*), and 2 (*yes*). We computed a weighted sum score according to the scale instructions (Babor et al., 2001).

### Statistical Analysis

We used the statistical software R and RStudio (R Development Core Team, 2019; RStudio Team, 2020) for reliability, descriptive, and correlation analyses. Internal consistencies were computed with the splithalf() function of the psych() package (Revelle, 2019). Holm-corrected Pearson correlations and *p* values were computed with the correlation() package (Makowski et al., 2020). We computed structural equation models using M*plus*8 (Muthen & Muthen, 2017). We used the M*plus*Automation() package to use M*plus* through RStudio (Hallquist & Wiley, 2013). We used the full information maximum likelihood procedure to deal with missing data. All models were estimated with the MLR estimator. We calculated intraclass correlations (ICC) for all variables to estimate the proportion of variance that can be attributed to similarities between siblings. Family membership accounted for 0.041 (sexual abuse) to 0.412 (physical abuse) of the variance (Supplementary Material S1 for detailed results). Thus, to account for the clustering of participants in families, we used the complex command in M*plus* and family membership as the cluster variable and repeated all analyses with a sample including only one random member per family.

The latent factors for sexual abuse, callous-unemotional traits, ASF, alcohol use, and rape-supportive attitudes were indicated by test halves consisting of the odd and the even items of the accordant measures, respectively. The latent factor for physical and verbal abuse was indicated by the physical- and the emotional-abuse subscale from the CTQ. Antisocial behavior/aggression was indicated by the mean scores of antisocial behavior and aggressive behavior. Sexual coercion was indicated by the single item on the use of force and the mean score reflecting the number of coercive strategies and the intensity of potentially resulting coercive acts. Pornography consumption and distorted beliefs were measured via only one item, respectively, and entered as manifest variables. All indicators loaded significantly on their respective factors. In order to test a structural equation model, the underlying measurement model needs to be overidentified. This is the case when each latent factor is indicated by at least two observed variables and when there are more than two latent factors (Wang & Wang, 2020). This was the case for all latent variables in our models. In addition, under this condition, two indicators are generally considered sufficient to reflect a latent variable (Wang & Wang, 2020). We tested a sequence of models. The first model (Model 1) replicated the model suggested by Knight and Sims-Knight (2011), that is, only estimating the paths described above and shown in Fig. 1. The second model estimated all potential indirect and direct effects within this model (Model 2). The third model included the additional variables as suggested by Knight and Sims-Knight (2011) except alcohol consumption and only estimated the paths described above and outlined in Fig. 1 (Model 3). The fourth model estimated all potential direct and indirect effects using the variables in Model 3 (Model 4). Because a model also including the interaction between antisocial behavior/aggression and alcohol consumption did not converge (presumably to the high complexity of the model), we estimated the assumed direct and interaction effect of alcohol consumption in a separate model that only included antisocial behavior/aggression, alcohol consumption, and sexual coercion (Model 5). We estimated the interaction effect via Monte Carlo simulations based on 1000 iterations. To determine the validity and robustness of our results, we calculated all models with each of the two ASF indicators as single manifest variables. Because the *χ*^2^-test is sensitive to sample size, model fits were considered acceptable if absolute fit indices were CFI ≥ 0.95, RMSEA ≤ 0.08, SRMR ≤ 0.05 (Hu & Bentler, 1999). In order to estimate the robustness of the derived effects, we computed confidence intervals based on the standard errors of the estimated effects via the tidySEM() package (Van Lissa, 2019). Model comparisons between the nested model 1 and model 2 as well as model 3 and model 4 were conducted via χ^2^-difference tests. Model comparisons between model 1 and model 3 as well as model 2 and model 4 were based on the BIC (Bayesian Information Criterion) scores (absolute difference in BIC 0–2 weak evidence, 2–6 positive evidence, 6–10 strong evidence, 10 + very strong evidence for the model with smaller values; Wang & Wang, 2020).

## Results

### Descriptive Statistics

Table 1 shows the internal consistencies, means, and standard deviations of all study variables. Almost all study variables showed small to medium positive correlations (Table 2). Only childhood sexual abuse was unrelated to almost all other variables in the study, verbal abuse was unrelated to sexual desire and sex drive, and physical abuse was unrelated to sexual desire.

**Table 1:** Descriptive statistics of all observed measures in the present study

Variables	*α*	*N*	Min	Max	*M*	*SD*
Frequency of sexual coercion	–	3110	0.00	0.50	1.18	0.57
Variety of coercive strategies and intensity of coercive acts	0.56	3021	1.00	5.00	0.02	0.05
Aggressive sexual fantasy	0.68	3071	− 1.35^a^	3.59^a^	− 0.0001^a^	0.87^a^
Callousness/unemotionality	0.64	3038	1.00	4.78	2.29	0.50
Antisocial behavior	0.80	3043	1.00	4.80	1.92	0.63
Aggression	0.85	2994	1.07	4.71	2.06	0.51
Sexual desire	0.76	3117	1.00	8.83	5.41	1.20
Sex drive	0.75	2855	0.00	7.83	3.18	0.90
Physical abuse	0.72	3217	1.00	5.00	1.35	0.49
Emotional abuse	0.78	3207	1.00	5.00	1.39	0.54
Sexual abuse	0.78	3212	1.00	4.20	1.04	0.19
Distorted perceptions	−	3076	0.00	10.00	1.53	1.49
Violent pornography consumption	−	3072	0.00	1.00	0.24	0.42
Rape-supportive attitudes	0.81	3065	1.00	7.00	1.61	1.11
Alcohol consumption	0.84	2811	1.00	36.00	10.25	6.13

^a^Based on *z*-standardized items

**Table 2:** Zero-Order Pearson correlations between all observed variables

	2	3	4	5	6	7	8	9	10	11	12	13	14	15
1 Frequency of sexual coercion	.340***	.187***	.120***	.174***	.096***	.106***	.128***	.101***	.099***	.106***	.088***	.105***	.148***	.114***
2 Variety of coercive strategies		.235***	.132***	.251***	.195***	.123***	.128***	.130***	.138***	.121***	.117***	.122***	.229***	.224***
3 Aggressive sexual fantasy			.162***	.144***	.157***	.215***	.134***	.058*	.103***	.095***	.391***	.449***	.485***	.113***
4 Callousness/unemotionality				.432***	.319***	.055*	.105***	.111***	.077***	.021	.146***	.108***	.183***	.108***
5 Antisocial behavior					.553***	.185***	.236***	.270***	.187***	.045	.115***	.112***	.171***	.476***
6 Aggression						.114***	.155***	.241***	.187***	.051	.158***	.083***	.199***	.337***
7 Sexual desire							.408***	.024	.025	.020	.071**	.162***	.163***	.200***
8 Sex drive								.075**	.022	-.005	.082***	.134***	.090***	.186***
9 Physical abuse									.539***	.171***	.065**	.017	.075***	.166***
10 Verbal abuse										.235***	.083***	.062**	.079***	.163***
11 Sexual abuse											.044	.036	.097***	.048
12 Distorted perceptions												.199***	.318***	.064*
13 Violent pornography use													.263***	.090***
14 Rape-supportive attitudes														.110***
15 Alcohol consumption														

*p *value adjustment method: Holm (1979); ***p < .001; **p < . 01; *p < . 05

### Replication of the Multifactorial Model of Sexual Aggression

**Fig. 2:**
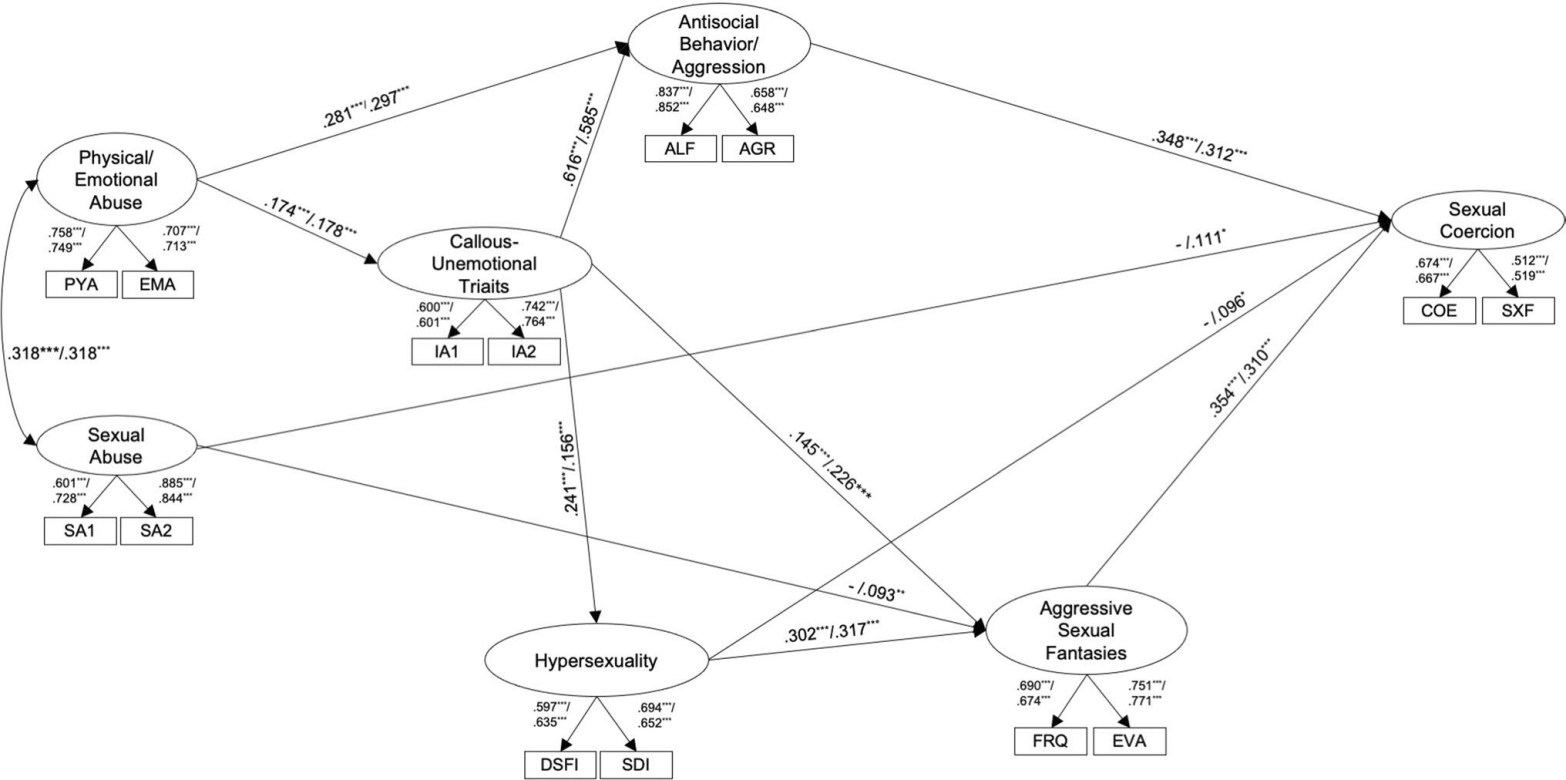
Results for the Replication of the Original/Initial Multifactor Model (Model 1) and the Inspection of All Possible Direct and Indirect Pathways (Model 2). *Note.* Predicting sexual coercion: Model 1 (*χ*^2^ = 303.674, *df* = 66, *p* < .001; RMSEA = .033 [.029; .037]; CFI = .959; SRMR = .036; *N* = 3269; *R*^2^ in ASF = .154, *p* < .001; *R*^2^ in sexual coercion = .296, *p* < .001). Only significant pathways displayed. / Model 2 (*χ*^2^ = 165.942, *df* = 56, *p* < .001; RMSEA = .025 [.020; .029]; CFI = .981; SRMR = .020; *N* = 3269; *R*^2^ in ASF = .167, *p* < .001; *R*^2^ in sexual coercion = .320, *p* < .001). All pathways allowed and estimated in Model 2. Only significant pathways displayed. SA1 = Mean of odd Sexual Abuse items; SA2 = Mean of even Sexual Abuse items; IA1 = Mean of odd Interpersonal/Affective Items; IA2 = Mean of even Interpersonal/Affective items; ALF = Antisocial Lifestyle; AGR = Aggression; DSFI = Derogatis Sexual Functioning Inventory; SDI = Sex Drive Inventory; FRQ = Frequency of aggressive sexual fantasies; EVA = Evaluation of aggressive sexual fantasies; COE = Versatility of coercive strategies and intensity of coercive acts; SXF = used sexual force; ^***^p < .001; ^**^p < . 01; ^*^p < . 05. – indicates that the path was not included in the respective model. All path coefficients can be seen in Supplementary Tables S1 and S2

The model for the conceptual replication of the original multifactor model (Model 1) explained 29.6% of the variance in sexual coercion and 15.4% of the variance in ASF (*χ*^2^ = 303.674, *df* = 66, *p* < 0.001; RMSEA = 0.033 [0.029; 0.037]; CFI = 0.959; SRMR = 0.036; *N* = 3269). In line with previous findings and Hypothesis 1, physical and emotional abuse during childhood was associated with callous-unemotional traits and antisocial behavior/aggression. Callous-unemotional traits were positively associated with antisocial behavior/aggression, hypersexuality, and ASF. Hypersexuality was positively associated with ASF. ASF and antisocial behavior/aggression were positively associated with sexual coercion. As in some of the previous studies, there neither was an association between sexual abuse during childhood and hypersexuality nor between antisocial behavior/aggression and ASF (Fig. 2 for significant path-coefficients, Table S2 in Supplementary Material for all path-coefficients).

We then inspected the same model with all potential direct and indirect effects allowed and estimated (Model 2). This model explained 32.0% of the variance in sexual coercion and 16.7% of the variance in ASF. This Model 2 showed a better fit with the data than Model 1 including only specified paths (*χ*^2^ = 165.942, *df* = 56, *p* < 0.001; RMSEA = 0.025 [0.020; 0.029]; CFI = 0.981; SRMR = 0.020; *N* = 3269; Δ*χ*^2^= 128.118, Δ*df* = 10, Δ*p* < 0.001). The previous result pattern persisted, but there were additional positive associations between sexual abuse during childhood and ASF, sexual abuse and sexual coercion, as well as hypersexuality and sexual coercion. Furthermore, there were significant indirect effects between physical and emotional abuse as well as callous-unemotional traits and sexual coercion via antisocial behavior/aggression, respectively. In addition, there was an indirect effect between callous-unemotional traits and sexual coercion via hypersexuality. Finally, there were indirect effects between sexual abuse during childhood, hypersexuality, as well as callous-unemotional traits and sexual coercion via ASF, respectively. (Fig. 2 for significant path-coefficients, Tables S3 and S6 in Supplementary Material for all path-coefficients and all significant indirect effects).

### The Extension of the Multifactorial Model of Sexual Aggression

**Fig. 3:**
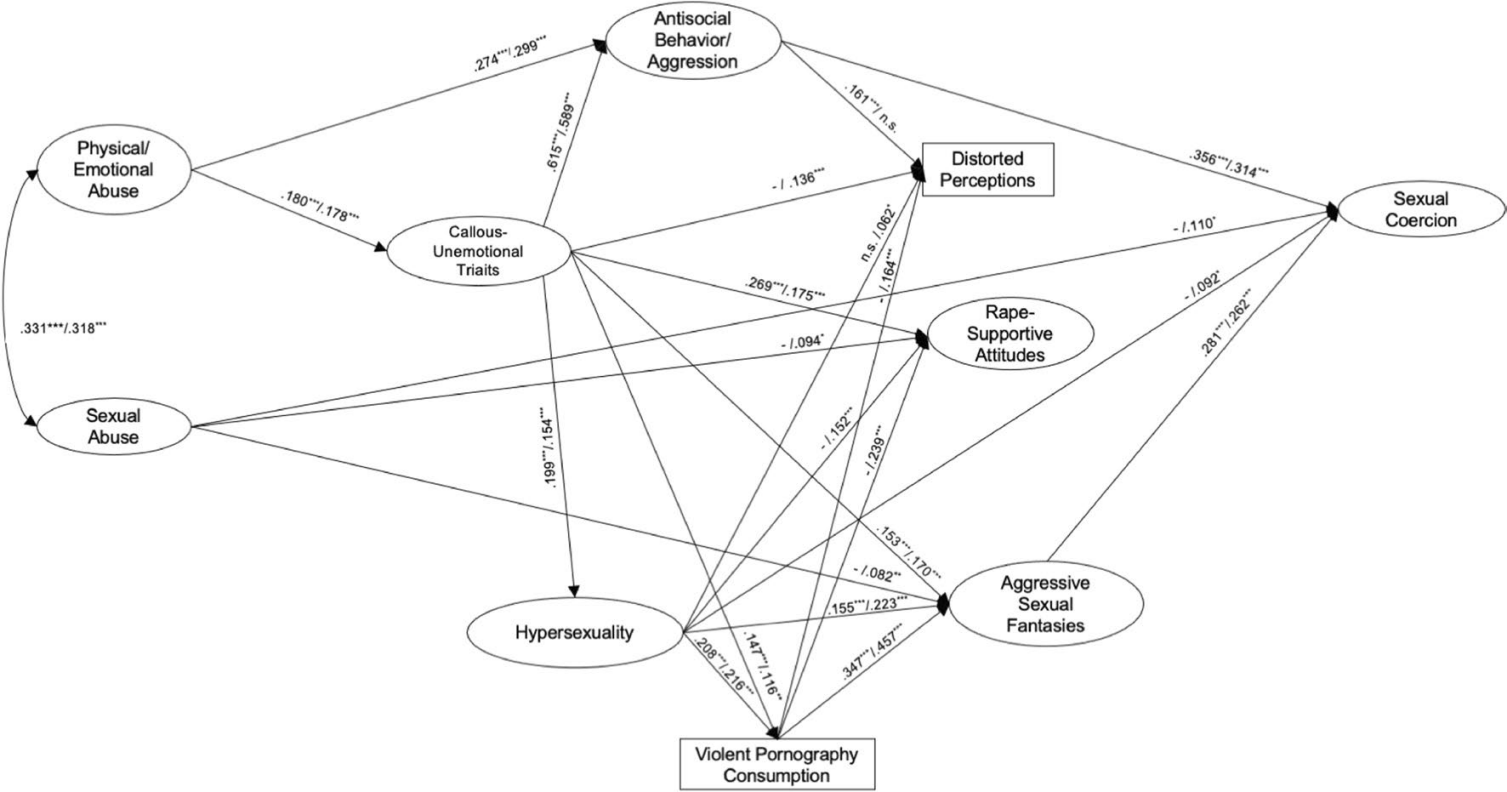
The Extension of the Multifactor Model (Model 3) and the Exploration of All Possible Pathways (Model 4). *Note*. Predicting sexual coercion: Model 3 (*χ*^2^ = 594.477, *df* = 113, *p* < .001; RMSEA = .036 [.033; .039]; CFI = .947; SRMR = .046; *N* = 3269; *R*^2^ in ASF = .231, *p* < .001; *R*^2^ in sexual coercion = .280, *p* < .001). Only significant pathways displayed. / Model 4 (*χ*^2^ = 300.433, *df* = 92, *p* < .001; RMSEA = .026 [.023; .030]; CFI = .977; SRMR = .020; *N* = 3269; *R*^2^ in ASF = .369, *p* < .001; *R*^2^ in sexual coercion = .323, *p* < .001). Only significant pathways displayed.; ^***^p < .001; ^**^p < . 01; ^*^p < . 05. Items for latent variables not depicted. – indicates that the path was not included in the respective model; All path coefficients can be seen in Supplementary Tables S3 and S4

When including distorted perceptions, rape-supportive attitudes, and violent pornography consumption into the original model (Model 1) and estimating the path-coefficients suggested by Knight and Sims-Knight (2011), this Model 3 explained 28.0% of the variance in sexual coercion and 23.1% of the variance in ASF (*χ*^2^ = 594.477, *df* = 113, *p* < 0.001; RMSEA = 0.036 [0.033; 0.039]; CFI = 0.947; SRMR = 0.046; *N* = 3269). Contrasting Hypothesis 2a, the model fits the data worse than Model 1 (BIC_Model 1_ = 48,283.74, BIC_Model 3_ = 78,872.79), but the result pattern of the initial results remained stable. Regarding the additional variables in the model, as hypothesized by the authors (Knight & Sims-Knight, 2011), hypersexuality was positively associated with violent pornography consumption, violent pornography consumption was positively associated with ASF, callous-unemotional traits were positively associated with rape-supportive attitudes, and antisocial behavior/aggression was positively associated with distorted perceptions. Contrasting the assumptions, hypersexuality was unrelated to distorted perceptions, and rape-supportive attitudes as well as distorted perceptions were unrelated to sexual coercion. Thus, the additional variables did not significantly add to the explanation of variance in sexual coercion (Fig. 3 for significant path-coefficients, Table S4 in Supplementary Material for all path-coefficients).

We then inspected the extended Model 3 with all potential direct and indirect effects between variables allowed and estimated (Model 4). This Model 4 explained 32.5% of the variance in sexual coercion and 36.9% of the variance in ASF. The model fit the data substantially better than Model 3 without the additional pathways (*χ*^2^ = 300.433, *df* = 92, *p* < 0.001; RMSEA = 0.026 [0.023; 0.030]; CFI = 0.977; SRMR = 0.020; *N* = 3269; Δ*χ*^2^ = 253.1866, Δ*df* = 12, Δ*p* < 0.001). The previous pattern of the results in Model 3 remained stable, but there were additional direct effects: Violent pornography consumption, hypersexuality, and callous-unemotional traits were positively associated with rape-supportive attitudes and distorted perceptions, respectively. Sexual abuse during childhood was also positively associated with rape-supportive attitudes. In line with the model assumptions, violent pornography consumption was positively associated with ASF. There were also indirect effects between sexual abuse as well as violent pornography and sexual coercion via ASF, respectively. Rape-supportive attitudes, distorted perceptions, and violent pornography consumption did not directly add to the explanation of variance in sexual coercion and there were also no indirect effects of rape-supportive attitudes and distorted perceptions (Fig. 3 for significant path-coefficients, Tables S5 and S6 in Supplementary Material for all path coefficient and significant indirect effects). The smaller BIC score of Model 2 in comparison with Model 4 (BIC_Model 2_ = 48,178.29, BIC_Model 4_ = 78,653.29) also indicated that the more parsimonious model excluding the additional variables should be favored.

Model 5 separately inspected the associations of alcohol consumption with sexual coercion also when interacting with antisocial behavior/aggression. There were positive associations of both antisocial behavior/aggression and alcohol consumption (*β* = 0.115,* p* = 0.004) with sexual coercion, but no significant interaction effect.

Further exploratory analyses showed that the pattern of relations with sexual coercion remained very similar in both, zero-order correlations and structural equation models, when only one of the two ASF indicators was considered (Supplementary Material S8-15). There, however, were notable differences in the prediction of these indicators: Callous/unemotional traits and sexual abuse consistently predicted a positive evaluation of ASF, but not ASF frequency; rape-supportive attitudes were an additional predictor of sexual coercion (all other relations remained stable as compared to the models using the latent ASF factor). When we repeated all models with a sample of one random member from each family, the overall pattern of results remained stable. However, physical abuse experiences predicted hypersexuality across all model iterations and rape-supportive attitudes predicted sexual coercion in model 3 using the latent ASF factor (Supplementary Material S16-19).

## Discussion

The present cross-sectional study aimed to examine the incremental validity of ASF for sexual aggression in a broad framework of other relevant risk factors and to replicate and extend a multifactor model for sexual aggression (Knight & Sims-Knight, 2004, 2006) that includes ASF as an important associate of this behavior. We could replicate the model in a large sample of men from the general population. Including all possible pathways between the initial variables improved the model fit and increased the amount of explained variance in sexual coercion, especially through the direct positive associations between sexual abuse during childhood as well as hypersexuality and sexual coercion. Including distorted perceptions, rape-supportive attitudes, and violent pornography consumption did not increase the amount of explained variance, indicating few or small effects of specific, facilitating risk factors for sexual coercion above and beyond more general, motivating factors and ASF. There was a positive association between alcohol consumption and sexual coercion in a reduced model. Antisocial behavior/aggression and ASF were the strongest associates of sexual coercion in all models. In addition, ASF mediated the associations between sexual coercion and many other variables in the study, providing evidence for its relevance for this behavior. The only small to moderate amount of variance that all other risk factors explained in ASF supports the notion that they should be considered as an independent correlate of sexual coercion.

### Aggressive Sexual Fantasies as a Potential Risk Factor for Sexual Coercion

Supporting previous research (Bondü & Birke, 2020, 2021; Knight & Sims-Knight, 2004, 2006), ASF were consistently associated with sexual coercion even when a broad range of other important cognitive and behavioral risk factors was considered, suggesting high incremental validity. The importance of ASF was underscored by the stability of its positive associations with sexual coercion irrespective of which additional pathways and variables were entered into the model, by showing path coefficients comparable in size with antisocial behavior/aggression, and by mediating the effects of sexual abuse experiences, hypersexuality, callous-unemotional traits, and violent pornography consumption on sexual coercion. Robust associations with sexual coercion when repeating the analyses with the single ASF items, respectively, underline the validity of both indicators and supports the assumption that various ASF qualities should be considered (Bartels & Beech, 2016; Bondü, 2023; Renaud & Byers, 1999). Future research may address closer associations of callous/unemotional traits and sexual abuse with ASF evaluation than with ASF frequency that indicate differential influences on ASF characteristics (e.g., individuals with callous/unemotional traits may find ASF more tempting or experience less guilt).

The amount of explained variance in ASF was moderate (36.9% maximum), indicating that ASF are in part, but not sufficiently explained by general, underlying risk factors motivating toward sexual aggression, such as antisocial traits and behavior or hypersexuality, nor by more specific factors that may facilitate this behavior, such as violent pornography consumption. Thus, these findings underscore the notion that ASF should be considered as an independent risk factor for sexual aggression that may motivate toward and facilitate accordant behavior at the same time.

This assumption is also supported by high zero-order correlations between ASF and risk factors that are thought to facilitate sexual aggression, that is, distorted beliefs, rape-supportive attitudes, and violent pornography consumption, supporting previous research (Bartels & Gannon, 2009; Bartels et al., 2020; Bondü & Birke, 2021; Dean & Malamuth, 1997). The lack of an increase in explained variance in sexual coercion when including these variables into the model suggests that ASF may explain the variance that would otherwise be accounted for by these variables (Abbey et al., 2011; Malamuth et al., 2021). This finding suggests that ASF may not only elaborate the scripts for executing this behavior (Huesmann, 1998), thereby pressuring toward their execution. They may also integrate existing justifications for sexual aggression (Marshall & Marshall, 2000) or press for the development of such justifications in order to diminish cognitive dissonance and to allow for following up on these fantasies, thereby further lowering the thresholds for acting upon them. Future research should further examine this notion.

In the present study, antisocial behavior/aggression and ASF showed similar associations with sexual coercion with path-coefficients of about *ß* = 0.35. That is, path-coefficients for ASF were somewhat smaller than in previous research (Knight & Sims-Knight, 2004, 2006). This may be the case, because previous research included sexual sadism as an indicator of ASF. Thus, in previous research, this factor not only included fantasies but potentially also past experiences of actual sexually sadistic behavior (Knight & Sims-Knight, 2004). Therefore, there may have been stronger overlaps and a larger amount of shared variance between predictor and outcome variables in the previous research.

Because the present findings built on cross-sectional data, more research is needed to examine the extent to which ASF can be considered a causal risk factor for sexual coercion and to further disentangle its relations with other risk factors. For example, future research should investigate whether callous-unemotional traits, hypersexuality, and violent pornography consumption really precede ASF or are mere correlates and carve out overlaps with and differences from other cognitive risk factors, such as distorted beliefs, hostile attitudes against women, or sexual interest in aggression-related contents. Note that the present study cannot determine whether ASF should be considered as a risk factor separate from sexual interests in aggression-related contents, or whether ASF should be considered as an indicator and potentially a mediator of these interests.

### The Replication and Extension of the Multifactor Model for Sexual Aggression

Supporting previous research and Hypothesis 1, all model iterations in the present study showed a successful and robust conceptual replication of the multifactor model for sexual aggression (Knight & Sims-Knight, 2006, 2011). This included similarities in path-coefficients as well as the lack of an association between childhood sexual abuse and hypersexuality. In line with research on male adolescents convicted of sexual offenses (Knight & Sims-Knight, 2004), the present study also did not find an association between antisocial behavior/aggression and ASF. This may be the case because callous-unemotional traits are more closely associated with ASF than antisocial behavior/aggression, that may be more relevant for actual behavior outcomes (Seto, 2019). In addition, the overlap between ASF and antisocial behavior/aggression in the present study may be smaller than in previous research, because in the present study, ASF were not indicated by sexual sadism that potentially include behavioral aspects. Our findings also suggest that early abuse experiences are directly associated with sexual coercion rather than indirectly via increased hypersexuality. This underlines the influence of experiences of sexual abuse for similar own behavior (potentially via early learning experiences that created an association between sexual arousal and force) (Aebi et al., 2015; Forsman et al., 2015), but does not support the assumption that these experiences result in high sex drive or the tendency toward sexual arousal (which may have physiological causes rather than resulting from learning experiences). Direct associations between hypersexuality and sexual coercion indicate that this factor should be considered an independent risk factor for sexual coercion (Seto, 2019). These findings also highlight the benefit of examining all potential links between the variables in the multifactor model. As outlined above, adding the further variables into the model, in contrast, did not allow for a more precise explanation of variance in sexual coercion and, therefore, seems negligible in the context of the total model.

Of note, however, research has pointed to alternative association patterns between these variables: For example, alcohol consumption not only interacted with antisocial behavior/aggression, but also with hostility toward women or impersonal sex (Greene & Davis, 2011; Lisco et al., 2012), and mediated the influence of psychopathic traits (Abbey et al., 2011). Rape attitudes and distorted beliefs may precede (Marshall & Marshall, 2000) or follow from (see above) ASF instead of being fairly independent. Further relevant variables that should be considered, for example, include the influence of peers and peer norms (Abbey et al., 2006; Jacques-Tiura et al., 2015; Malamuth et al., 2021). Finally, the present reasoning and recent research suggest bi-directional associations between many study variables (Stefanska et al., 2022) that may be better captured by a network approach (Smid et al., 2024; Van Den Berg et al., 2023).

Replicating and extending the model in a large community sample points to the potential to establish a “unified framework” for sexual aggression that explains this behavior in forensic and community contexts alike and thereby stronger connects social, clinical, and forensic psychology research (Knight & Sims-Knight, 2006; Malamuth, 2006). This is particularly interesting because previous models often used operationalizations that were tailored to forensic samples, whereas the present measures were suited for non-clinical samples. For example, we used a new factor solution for the psychopathy measure and narrow indicators for the ASF factor, reducing overlaps between antisocial behavior/aggression, ASF, and sexual coercion by relinquishing items that all assess past sexual aggression, respectively (Dotterer et al., 2017; Hollerbach et al., 2018). Thus, the present study has the advantage of using alternative operationalizations of the variables in the original studies. In some cases, however, the broad spectrum of contents covered by single factors in the original model was not as comprehensively or somewhat differently represented in the present study (e.g., the factor callous-unemotional traits originally also included aspects of hostile masculinity that could not be considered in the present study; non-sexual abuse experiences were originally indicated by physical and emotional rather than by physical and verbal abuse).

### Limitations and Outlook

The strengths of the present study include the large sample size and the comprehensive replication and extension of the model in the general population by an independent team of researchers. Limitations include the cross-sectional data that prevents causal inferences. For example, the tendency to often experience ASF may also predict hypersexuality. Even memories of adverse childhood experiences may be distorted by subsequent own behavior. The data are not representative because they comprised a large number of twins and siblings and strongly rely on self-reports. Distorted perceptions, rape-supporting attitudes, violent pornography consumption, and ASF were measured with small numbers of items that may not represent all relevant aspects of these variables and that limit the reliability and validity of these measures and the comparability with previous research on the multifactor model. Low internal consistencies of some of the present variables may limit the generalizability of the present findings as well. Note, however, that although some internal consistencies seem low at first sight, they often reflect intercorrelations of only two items (e.g., ASF) as well as different behaviors that do not necessarily have to go along with one another (e.g., different coercive strategies). In addition, for example, both ASF indicators showed similar association patterns with the outcomes, further lending support to the reliability of the findings. We indicated hypersexuality via measures for high sex drive and desire and, thus, captured one subclinical facet of hypersexuality, but not sexual preoccupation and compulsivity as in previous studies. The item to assess distorted perceptions intended to capture an assessment of the subjective proportion of women who like to be sexually coerced. However, the item can also be read in a way that it would ask for the assessment of the proportion of women who like to sexually coerce someone themselves. Finally, the items on alcohol consumption measured its general frequency and the negative consequences of drinking, rather than alcohol consumption specifically in situations when sexually coercive behavior occurred. Taken together, future research should adapt more comprehensive and suitable measures for these constructs (Abbey et al., 2022; Bondü & Birke, 2021; Bőthe et al., 2018; Bumby, 1996; Malamuth et al., 2021). This will also allow for separately considering different ASF contents (Bondü & Birke, 2021) and levels of elaboration (Willis & Bartels, 2022). Due to convergence problems, we were unable to include alcohol consumption into the total model. The original extension of the multifactor model suggests to consider genetic influences that were beyond the scope of the present study. In addition, further potential risk factors for sexual coercion, such as further facets of hostile masculinity or aggression-related sexual interests, were not considered in the model. Future research may strive to use longitudinal data, more comprehensive measures, and additional information about potential genetic influences in order to replicate the model and investigate potential additional variables and interaction effects.

### Conclusion

The present study successfully replicated and extended an important multifactor model of sexual aggression in a large community sample, providing further empirical evidence for a unified model of sexual aggression that may be relevant for both forensic and community samples. It contributes to a better understanding of the complex interplay of risk factors for sexual aggression and highlights the central role of ASF. Thus, ASF should be considered in both forensic and clinical settings when evaluating the individual risk of sexual aggressive behavior and in developing targeted intervention and prevention measures (Allen et al., 2020, for an overview). Recent research suggested that sexual fantasies in general can be altered by bilateral stimulation via eye-movements effecting their appraisal and their frequency (Allen et al., 2023; Bartels et al., 2018), which potentially complements other intervention efforts, such as reconditioning procedures (Rossegger et al., 2021, for an overview). Given that ASF are considered a central mediator, it seems reasonable to target potential preceding risk factors: For example, interventions that tackle hypersexual behavior or excessive pornography consumption by increasing sexual self-control may reduce the frequency of problematic sexual fantasies (Briken, 2020). Sex-education programs should address potential influences of violent pornography consumption on sexual fantasizing and related sexual scripts and promote a critical appraisal of pornographic contents in order to decrease the likelihood that violent elements are incorporated into sexual fantasizing (Wright, 2014). In addition, the knowledge about relevant risk factors, including those that cannot easily be altered, such as callous-unemotional traits, as well as the interplay between these factors may inform practitioners’ evaluation of the risk for re-offending (Mann et al., 2010). Thus, the present study underscores that ASF deserve attention in risk assessment of sexual violence and parole decisions.

### Supplementary Information

Below is the link to the electronic supplementary material.

Supplementary file1 (DOCX 112 kb)

## Data Availability

Data will be made available upon request.

## Code availability

Analysis script is available at https://osf.io/4rtdu/
