# Supplementary material for: Links between Aggressive Sexual Fantasies and Sexual Coercion: A Replication and Extension of a Multifactorial Model
Source: Arch Sex Behav. 2024 Jan 17;53(3):1047–63. doi: 10.1007/s10508-023-02782-5 (PMC10920420; doi:10.1007/s10508-023-02782-5)
Supplement: Supplementary file 1 — Supplementary file1 (DOCX 112 kb) [file 10508_2023_2782_MOESM1_ESM.docx]

Links between Aggressive Sexual Fantasies and Sexual Coercion: A Replication and Extension of a Multifactorial Model

Supplementary Material

Table S1

*Intraclass-Correlation Coefficients for all Manifest Variables*

| Manifest Variables | ICC |
| --- | --- |
| Frequency of Sexual Coercion | .065 |
| Variety of Coercive Strategies | .057 |
| Aggressive Sexual Fantasy | .238 |
| Callousness/Unemotionality | .178 |
| Antisocial Behavior | .396 |
| Aggression | .273 |
| Sexual Desire | .226 |
| Sex Drive | .193 |
| Physical Abuse | .412 |
| Verbal Abuse | .347 |
| Sexual Abuse | .041 |
| Distorted Perceptions | .093 |
| Violent Pornography Use | .171 |
| Rape-Supportive Attitudes | .112 |
| Alcohol Consumption | .403 |

Table S2

*Model 1: Replication of the Original Model. Standardized Estimates.*

| Factors | β | *SE* | 95% CI |
| --- | --- | --- | --- |
| Physical and Emotional Abuse 🡪 Antisocial Behavior | 0.28^***^ | 0.03 | [0.22, 0.34] |
| Physical and Emotional Abuse 🡪 Callous-Unemotional Traits | 0.17^***^ | 0.03 | [0.12, 0.23] |
| Sexual Abuse 🡪 Hypersexuality | 0.02 | 0.03 | [-0.04, 0.08] |
| Callous-Unemotional Traits 🡪 Antisocial Behavior | 0.62^***^ | 0.03 | [0.56, 0.67] |
| Callous-Unemotional Traits 🡪 Aggressive Sexual Fantasies | 0.14^**^ | 0.05 | [0.05, 0.24] |
| Callous-Unemotional Traits 🡪 Hypersexuality | 0.24^***^ | 0.04 | [0.16, 0.32] |
| Antisocial Behavior 🡪 Sexual Coercion | 0.35^***^ | 0.03 | [0.29, 0.41] |
| Antisocial Behavior 🡪 Aggressive Sexual Fantasies | 0.06 | 0.04 | [-0.03, 0.15] |
| Hypersexuality 🡪 Aggressive Sexual Fantasies | 0.30^***^ | 0.03 | [0.25, 0.36] |
| Aggressive Sexual Fantasies 🡪 Sexual Coercion | 0.35^***^ | 0.04 | [0.29, 0.42] |
|  | *r* | *SE* | 95% CI |
| Sexual Abuse ↔ Physical Abuse | 0.32^***^ | 0.04 | [0.24, 0.40] |

*Note.* Indirect effects are shown in Table S6 below. ^***^*p*<.001; ^**^*p*<.01; ^*^*p*<.05

Table S3

*Model 2: Original Model with All Pathways Allowed and Estimated. Standardized Estimates.*

| Factors | β | *SE* | 95% CI |
| --- | --- | --- | --- |
| Physical Abuse 🡪 Antisocial Behavior | 0.30^***^ | 0.03 | [0.23, 0.36] |
| Physical Abuse 🡪 Callous-Unemotional Traits | 0.18^***^ | 0.03 | [0.11, 0.24] |
| Physical Abuse 🡪 Sexual Coercion | 0.06 | 0.05 | [-0.03, 0.15] |
| Physical Abuse 🡪 Aggressive Sexual Fantasies | 0.06 | 0.04 | [-0.01, 0.13] |
| Physical Abuse 🡪 Hypersexuality | 0.05 | 0.03 | [-0.02, 0.11] |
| Sexual Abuse 🡪 Antisocial Behavior | -0.05 | 0.03 | [-0.10, 0.00] |
| Sexual Abuse 🡪 Callous-Unemotional Traits | -0.03 | 0.03 | [-0.09, 0.03] |
| Sexual Abuse 🡪 Sexual Coercion | 0.11^*^ | 0.05 | [0.01, 0.21] |
| Sexual Abuse 🡪 Aggressive Sexual Fantasies | 0.09^**^ | 0.03 | [0.03, 0.15] |
| Sexual Abuse 🡪 Hypersexuality | -0.01 | 0.03 | [-0.08, 0.05] |
| Callous-Unemotional Traits 🡪 Antisocial Behavior | 0.58^***^ | 0.03 | [0.53, 0.64] |
| Callous/Unemotional Traits 🡪 Sexual Coercion | -0.04 | 0.05 | [-0.14, 0.07] |
| Callous-Unemotional Traits 🡪 Aggressive Sexual Fantasies | 0.23^***^ | 0.04 | [0.14, 0.31] |
| Callous-Unemotional Traits 🡪 Hypersexuality | 0.16^***^ | 0.03 | [0.09, 0.22] |
| Antisocial Behavior 🡪 Sexual Coercion | 0.31^***^ | 0.06 | [0.19, 0.43] |
| Antisocial Behavior 🡪 Aggressive Sexual Fantasies | -0.07 | 0.05 | [-0.17, 0.03] |
| Hypersexuality 🡪 Sexual Coercion | 0.10^*^ | 0.04 | [0.02, 0.17] |
| Hypersexuality 🡪 Aggressive Sexual Fantasies | 0.32^***^ | 0.03 | [0.25, 0.38] |
| Aggressive Sexual Fantasies 🡪 Sexual Coercion | 0.31^***^ | 0.04 | [0.23, 0.39] |
|  | *r* | *SE* | 95% CI |
| Sexual Abuse ↔ Physical Abuse | 0.33^***^ | 0.04 | [0.25, 0.42] |
| Hypersexuality ↔ Antisocial Behavior | 0.35^***^ | 0.04 | [0.28, 0.42] |

*Note.* Indirect effects are shown in Table S6 below. ^***^*p*<.001; ^**^*p*<.01; ^*^*p*<.05

Table S4

*Model 3: Extended Model. Standardized Estimates.*

| Factors | β | *SE* | 95% CI |
| --- | --- | --- | --- |
| Physical Abuse 🡪 Antisocial Behavior | 0.27^***^ | 0.03 | [0.22, 0.33] |
| Physical Abuse 🡪 Callous-Unemotional Traits | 0.18^***^ | 0.03 | [0.12, 0.24] |
| Sexual Abuse 🡪 Hypersexuality | 0.02 | 0.03 | [-0.04, 0.07] |
| Callous-Unemotional Traits 🡪 Antisocial Behavior | 0.61^***^ | 0.03 | [0.56, 0.67] |
| Callous-Unemotional Traits 🡪 Rape-Supportive Attitudes | 0.27^***^ | 0.03 | [0.21, 0.33] |
| Callous-Unemotional Traits 🡪 Aggressive Sexual Fantasies | 0.15^***^ | 0.04 | [0.08, 0.23] |
| Callous-Unemotional Traits 🡪 Violent Pornography Consumption | 0.15^***^ | 0.04 | [0.07, 0.22] |
| Callous-Unemotional Traits 🡪 Hypersexuality | 0.20^***^ | 0.03 | [0.13, 0.26] |
| Antisocial Behavior 🡪 Sexual Coercion | 0.36^***^ | 0.03 | [0.30, 0.42] |
| Antisocial Behavior 🡪 Distorted Perceptions | 0.16^***^ | 0.03 | [0.11, 0.21] |
| Antisocial Behavior 🡪 Aggressive Sexual Fantasies | 0.03 | 0.04 | [-0.05, 010] |
| Antisocial Behavior 🡪 Violent Pornography Consumption | -0.03 | 0.04 | [-0.11, 0.04] |
| Hypersexuality 🡪 Distorted Perceptions | 0.03 | 0.03 | [-0.02, 0.09] |
| Hypersexuality 🡪 Aggressive Sexual Fantasies | 0.15^***^ | 0.03 | [0.10, 0.21] |
| Hypersexuality 🡪 Violent Pornography Consumption | 0.21^***^ | 0.03 | [0.16, 0.26] |
| Distorted Perceptions 🡪 Sexual Coercion | -0.05 | 0.03 | [-0.11, 0.01] |
| Rape-Supportive Attitudes 🡪 Sexual Coercion | 0.08 | 0.05 | [-0.02, 0.19] |
| Aggressive Sexual Fantasies 🡪 Sexual Coercion | 0.28^***^ | 0.05 | [0.18, 0.39] |
| Violent Pornography Consumption 🡪 Aggressive Sexual Fantasies | 0.35^***^ | 0.03 | [0.30, 0.40] |
|  | *r* | *SE* | 95% CI |
| Sexual Abuse ↔ Physical Abuse | 0.32^***^ | 0.04 | [0.24, 0.40] |
| Hypersexuality ↔ Antisocial Behavior | 0.34^***^ | 0.04 | [0.27, 0.42] |
| Aggressive Sexual Fantasies ↔ Distorted Perceptions | 0.44^***^ | 0.03 | [0.39, 0.49] |
| Rape-Supportive Attitudes ↔ Aggressive Sexual Fantasies | 0.62^***^ | 0.03 | [0.56, 0.68] |
| Rape-Supportive Attitudes ↔ Distorted Perceptions | 0.30^***^ | 0.03 | [0.25, 0.35] |

*Note.* Indirect effects are shown in Table S6 below. ^***^*p*<.001; ^**^*p*<.01; ^*^*p*<.05

Table S5

*Model 4: Extended Model with All Pathways Allowed and Estimated. Standardized Estimates.*

| Factors | β | *SE* | 95% CI |
| --- | --- | --- | --- |
| Physical Abuse 🡪 Antisocial Behavior | 0.30^***^ | 0.03 | [0.24, 0.36] |
| Physical Abuse 🡪 Callous-Unemotional Traits | 0.18^***^ | 0.03 | [0.11, 0.24] |
| Physical Abuse 🡪 Distorted Perceptions | 0.06 | 0.03 | [-0.01, 0.12] |
| Physical Abuse 🡪 Sexual Coercion | 0.06 | 0.05 | [-0.03, 0.15] |
| Physical Abuse 🡪 Rape-Supportive Attitudes | 0.02 | 0.03 | [-0.04, 0.08] |
| Physical Abuse 🡪 Aggressive Sexual Fantasies | 0.06 | 0.03 | [-0.01, 0.12] |
| Physical Abuse 🡪 Violent Pornography Consumption | 0.02 | 0.03 | [-0.04, 0.07] |
| Physical Abuse 🡪 Hypersexuality | 0.05 | 0.03 | [-0.02, 0.11] |
| Sexual Abuse 🡪 Antisocial Behavior | -0.05 | 0.03 | [-0.10, 0.00] |
| Sexual Abuse 🡪 Callous-Unemotional Traits | -0.03 | 0.03 | [-0.09, 0.03] |
| Sexual Abuse 🡪 Distorted Perceptions | 0.02 | 0.03 | [-0.03, 0.07] |
| Sexual Abuse 🡪 Sexual Coercion | 0.11^*^ | 0.05 | [0.01, 0.21] |
| Sexual Abuse 🡪 Rape-Supportive Attitudes | 0.09^*^ | 0.04 | [0.02, 0.16] |
| Sexual Abuse 🡪 Aggressive Sexual Fantasies | 0.08^**^ | 0.03 | [0.03, 0.14] |
| Sexual Abuse 🡪 Violent Pornography Consumption | 0.03 | 0.02 | [-0.02, 0.07] |
| Sexual Abuse 🡪 Hypersexuality | -0.01 | 0.03 | [-0.07, 0.05] |
| Callous-Unemotional Traits 🡪 Antisocial Behavior | 0.59^***^ | 0.03 | [0.54, 0.64] |
| Callous-Unemotional Traits 🡪 Distorted Perceptions | 0.14^***^ | 0.04 | [0.06, 0.21] |
| Callous/Unemotional Traits 🡪 Sexual Coercion | -0.04 | 0.05 | [-0.15, 0.06] |
| Callous-Unemotional Traits 🡪 Rape-Supportive Attitudes | 0.18^***^ | 0.04 | [0.09, 0.26] |
| Callous-Unemotional Traits 🡪 Aggressive Sexual Fantasies | 0.17^***^ | 0.04 | [0.10, 0.25] |
| Callous-Unemotional Traits 🡪 Violent Pornography Consumption | 0.11^**^ | 0.04 | [0.04, 0.18] |
| Callous-Unemotional Traits 🡪 Hypersexuality | 0.15^***^ | 0.03 | [0.09, 0.22] |
| Antisocial Behavior 🡪 Sexual Coercion | 0.32^***^ | 0.06 | [0.20, 0.44] |
| Antisocial Behavior 🡪 Distorted Perceptions | 0.01 | 0.04 | [-0.08, 0.09] |
| Antisocial Behavior 🡪 Rape-Supportive Attitudes | 0.01 | 0.05 | [-0.09, 0.11] |
| Antisocial Behavior 🡪 Aggressive Sexual Fantasies | -0.05 | 0.04 | [-0.14, 0.03] |
| Antisocial Behavior 🡪 Violent Pornography Consumption | -0.03 | 0.04 | [-0.11, 0.06] |
| Hypersexuality 🡪 Distorted Perceptions | 0.06^*^ | 0.03 | [0.01, 0.12] |
| Hypersexuality 🡪 Sexual Coercion | 0.09^*^ | 0.04 | [0.01, 0.17] |
| Hypersexuality 🡪 Rape-Supportive Attitudes | 0.15^***^ | 0.03 | [0.09, 0.21] |
| Hypersexuality 🡪 Aggressive Sexual Fantasies | 0.22^***^ | 0.03 | [0.17, 0.28] |
| Hypersexuality 🡪 Violent Pornography Consumption | 0.22^***^ | 0.03 | [0.17, 0.27] |
| Violent Pornography Consumption 🡪 Distorted Perceptions | 0.16^***^ | 0.02 | [0.12, 0.21] |
| Violent Pornography Consumption 🡪 Rape-Supportive Attitudes | 0.24^***^ | 0.02 | [0.19, 0.28] |
| Violent Pornography Consumption 🡪 Sexual Coercion | -0.07 | 0.03 | [-0.13, 0.00] |
| Violent Pornography Consumption 🡪 Aggressive Sexual Fantasies | 0.46^***^ | 0.02 | [0.42, 0.51] |
| Distorted Perceptions 🡪 Sexual Coercion | -0.05 | 0.03 | [-0.11, 0.02] |
| Rape-Supportive Attitudes 🡪 Sexual Coercion | 0.05 | 0.05 | [-0.05, 0.16] |
| Aggressive Sexual Fantasies 🡪 Sexual Coercion | 0.34^***^ | 0.07 | [0.20, 0.47] |
|  | *r* | *SE* | 95% CI |
| Sexual Abuse ↔ Physical Abuse | 0.33^***^ | 0.04 | [0.25, 0.42] |
| Hypersexuality ↔ Antisocial Behavior | 0.35^***^ | 0.04 | [0.28, 0.42] |
| Aggressive Sexual Fantasies ↔ Distorted Perceptions | 0.42^***^ | 0.03 | [0.37, 0.47] |
| Rape-Supportive Attitudes ↔ Aggressive Sexual Fantasies | 0.59^***^ | 0.03 | [0.53, 0.65] |
| Rape-Supportive Attitudes ↔ Distorted Perceptions | 0.26^***^ | 0.03 | [0.21, 0.31] |

*Note.* Indirect effects are shown in Table S6 below. ^***^*p*<.001; ^**^*p*<.01; ^*^*p*<.05

Table S6

*Standardized Estimates of All Significant Indirect Effects on Sexual Coercion in Model 2 and 4.*

|  | **Model 2** | |  | **Model 4** | |
| --- | --- | --- | --- | --- | --- |
|  | β | *SE* |  | β | *SE* |
| Physical Abuse → Antisocial Behavior | .093^***^ | .020 |  | .094^***^ | .020 |
| Physical Abuse → Callous-Unemotional Traits → Hypersexuality | .003^*^ | .001 |  | n.s. | n.s. |
| Physical Abuse → Callous-Unemotional Traits → Antisocial Behavior | .032^***^ | .009 |  | .033^***^ | .009 |
| Physical Abuse → Callous-Unemotional Traits → Aggressive Sexual Fantasies | .012^**^ | .004 |  | .008^**^ | .003 |
| Physical Abuse → Callous-Unemotional Traits→ Hypersexuality → Aggressive Sexual Fantasies | .003^**^ | .001 |  | .002^**^ | .001 |
| Physical Abuse → Callous-Unemotional Traits → Violent Pornography Consumption → Aggressive Sexual Fantasies | - | - |  | .002^*^ | .001 |
| Physical Abuse → Callous-Unemotional Traits→ Hypersexuality → Violent Pornography Consumption → Aggressive Sexual Fantasies | - | - |  | .001^**^ | .000 |
|  |  |  |  |  |  |
| Sexual Abuse → Aggressive Sexual Fantasies | .029^**^ | .010 |  | .022^*^ | .009 |
|  |  |  |  |  |  |
| Callous-Unemotional Traits → Antisocial Behavior | .182^***^ | .037 |  | .185^***^ | .038 |
| Callous-Unemotional Traits → Hypersexuality | .015^*^ | .007 |  | .014^*^ | .007 |
| Callous-Unemotional Traits → Aggressive Sexual Fantasies | .070^***^ | .016 |  | .045^**^ | .015 |
| Callous-Unemotional Traits → Hypersexuality→ Aggressive Sexual Fantasies | .015^***^ | .004 |  | .009^**^ | .003 |
| Callous-Unemotional Traits→ Violent Pornography Consumption→ Aggressive Sexual Fantasies | - | - |  | .014^*^ | .005 |
| Callous-Unemotional Traits → Hypersexuality → Violent Pornography Consumption → Aggressive Sexual Fantasies | - | - |  | .004^**^ | .001 |
|  |  |  |  |  |  |
| Hypersexuality → Aggressive Sexual Fantasies | .098^***^ | .016 |  | .058^***^ | .015 |
| Hypersexuality → Violent Pornography Consumption→ Aggressive Sexual Fantasies | - | - |  | .026^***^ | .006 |
|  |  |  |  |  |  |
| Violent Pornography Consumption → Aggressive Sexual Fantasies | - | - |  | .120^***^ | .027 |

*Note.* ^***^*p*<.001; ^**^*p*<.01; ^*^*p*<.05

|  | **Model 1** | |  | **Model 2** | |  | **Model 3** | |  | **Model 4** | |
| --- | --- | --- | --- | --- | --- | --- | --- | --- | --- | --- | --- |
|  | β | *SE* |  | β | *SE* |  | β | *SE* |  | β | *SE* |
| *Physical/Emotional Abuse by…* |  |  |  |  |  |  |  |  |  |  |  |
| Mean of Emotional Abuse Items | 0.71^***^ | 0.03 |  | 0.71^***^ | 0.03 |  | 0.71^***^ | 0.03 |  | 0.71^***^ | 0.03 |
| Mean of Physical Abuse Items | 0.76^***^ | 0.03 |  | 0.75^***^ | 0.03 |  | 0.76^***^ | 0.03 |  | 0.75^***^ | 0.03 |
| *Sexual Abuse by…* |  |  |  |  |  |  |  |  |  |  |  |
| Mean of odd Sexual Abuse items | 0.69^***^ | 0.06 |  | 0.71^***^ | 0.03 |  | 0.69^***^ | 0.06 |  | 0.73^***^ | 0.06 |
| Mean of even Sexual Abuse items | 0.89^***^ | 0.06 |  | 0.75^***^ | 0.03 |  | 0.89^***^ | 0.06 |  | 0.84^***^ | 0.06 |
| *Callous-Unemotional Traits by…* |  |  |  |  |  |  |  |  |  |  |  |
| Mean of odd Interpersonal/Affective Items | 0.60^***^ | 0.02 |  | 0.60^***^ | 0.02 |  | 0.60^***^ | 0.02 |  | 0.61^***^ | 0.02 |
| Mean of even Interpersonal/Affective items | 0.74^***^ | 0.02 |  | 0.76^***^ | 0.02 |  | 0.74^***^ | 0.02 |  | 0.76^***^ | 0.02 |
| *Antisocial Behavior by…* |  |  |  |  |  |  |  |  |  |  |  |
| Aggression | 0.66^***^ | 0.02 |  | 0.65^***^ | 0.02 |  | 0.66^***^ | 0.02 |  | 0.65^***^ | 0.02 |
| Antisocial Lifestyle | 0.84^***^ | 0.02 |  | 0.85^***^ | 0.02 |  | 0.83^***^ | 0.02 |  | 0.85^***^ | 0.02 |
| *Hypersexuality by…* |  |  |  |  |  |  |  |  |  |  |  |
| Derogatis Sexual Functioning Inventory | 0.60^***^ | 0.04 |  | 0.64^***^ | 0.03 |  | 0.65^***^ | 0.03 |  | 0.62^***^ | 0.03 |
| Sex Drive Inventory | 0.69^***^ | 0.04 |  | 0.65^***^ | 0.03 |  | 0.64^***^ | 0.03 |  | 0.66^***^ | 0.03 |
| *Aggressive Sexual Fantasies by…* |  |  |  |  |  |  |  |  |  |  |  |
| Evaluation of ASF | 0.75^***^ | 0.03 |  | 0.77^***^ | 0.03 |  | 0.74^***^ | 0.02 |  | 0.76^***^ | 0.02 |
| Frequency of ASF | 0.69^***^ | 0.03 |  | 0.67^***^ | 0.03 |  | 0.66^***^ | 0.02 |  | 0.68^***^ | 0.02 |
| *Rape-Supportive Attitudes by…* |  |  |  |  |  |  |  |  |  |  |  |
| Likelihood to rape of best friend | / | / |  | / | / |  | 0.76^***^ | 0.02 |  | 0.75^***^ | 0.02 |
| Likelihood to Rape | / | / |  | / | / |  | 0.91^***^ | 0.02 |  | 0.92^***^ | 0.02 |
| *Sexual Coercion by…* |  |  |  |  |  |  |  |  |  |  |  |
| Versatility of coercive strategies and intensity of coercive acts | 0.67^***^ | 0.04 |  | 0.67^***^ | 0.04 |  | 0.68^***^ | 0.04 |  | 0.68^***^ | 0.04 |
| Used sexual force | 0.51^***^ | 0.03 |  | 0.52^***^ | 0.03 |  | 0.50^***^ | 0.03 |  | 0.51^***^ | 0.03 |

Table S7

*Standardized Factor Loadings for All Latent Variables*

*Note.* ^***^*p*<.001; ^**^*p*<.01; ^*^*p*<.05

Table S8

*Model 1: Replication of the Original Model Using the Frequency of ASF as Manifest Variable. Standardized Estimates.*

| Factors | β | *SE* | 95% CI |
| --- | --- | --- | --- |
| Physical and Emotional Abuse 🡪 Antisocial Behavior | 0.28^***^ | 0.03 | [0.23, 0.34] |
| Physical and Emotional Abuse 🡪 Callous-Unemotional Traits | 0.17^***^ | 0.03 | [0.11, 0.23] |
| Sexual Abuse 🡪 Hypersexuality | 0.01 | 0.03 | [-0.05, 0.07] |
| Callous-Unemotional Traits 🡪 Antisocial Behavior | 0.62^***^ | 0.03 | [0.57, 0.68] |
| Callous-Unemotional Traits 🡪 Frequency of ASF | 0.03 | 0.04 | [-0.04, 0.11] |
| Callous-Unemotional Traits 🡪 Hypersexuality | 0.25^***^ | 0.04 | [0.17, 0.33] |
| Antisocial Behavior 🡪 Sexual Coercion | 0.39^***^ | 0.03 | [0.33, 0.45] |
| Antisocial Behavior 🡪 Frequency of ASF | 0.07 | 0.04 | [-0.00, 0.14] |
| Hypersexuality 🡪 Frequency of ASF | 0.22^***^ | 0.03 | [0.17, 0.27] |
| Frequency of ASF 🡪 Sexual Coercion | 0.24^***^ | 0.03 | [0.19, 0.30] |
|  | *r* | *SE* | 95% CI |
| Sexual Abuse ↔ Physical Abuse | 0.32^***^ | 0.04 | [0.24, 0.40] |

*Note.* ^***^*p*<.001; ^**^*p*<.01; ^*^*p*<.05

Table S9

*Model 2: Model with All Pathways Allowed and Estimated Using the Frequency of ASF as Manifest Variable. Standardized Estimates.*

| Factors | β | *SE* | 95% CI |
| --- | --- | --- | --- |
| Physical Abuse 🡪 Antisocial Behavior | 0.30^***^ | 0.03 | [0.24, 0.36] |
| Physical Abuse 🡪 Callous-Unemotional Traits | 0.18^***^ | 0.03 | [0.11, 0.24] |
| Physical Abuse 🡪 Sexual Coercion | 0.07 | 0.05 | [-0.02, 0.17] |
| Physical Abuse 🡪 Frequency of ASF | 0.04 | 0.03 | [-0.02, 0.09] |
| Physical Abuse 🡪 Hypersexuality | 0.05 | 0.03 | [-0.02, 0.11] |
| Sexual Abuse 🡪 Antisocial Behavior | -0.05 | 0.03 | [-0.10, 0.00] |
| Sexual Abuse 🡪 Callous-Unemotional Traits | -0.03 | 0.03 | [-0.09, 0.03] |
| Sexual Abuse 🡪 Sexual Coercion | 0.13^*^ | 0.05 | [0.02, 0.23] |
| Sexual Abuse 🡪 Frequency of ASF | 0.04 | 0.03 | [-0.01, 0.09] |
| Sexual Abuse 🡪 Hypersexuality | -0.01 | 0.03 | [-0.08, 0.05] |
| Callous-Unemotional Traits 🡪 Antisocial Behavior | 0.58^***^ | 0.03 | [0.53, 0.63] |
| Callous/Unemotional Traits 🡪 Sexual Coercion | 0.01 | 0.05 | [-0.09, 0.11] |
| Callous-Unemotional Traits 🡪 Frequency of ASF | 0.08^*^ | 0.04 | [0.01, 0.15] |
| Callous-Unemotional Traits 🡪 Hypersexuality | 0.16^***^ | 0.03 | [0.09, 0.22] |
| Antisocial Behavior 🡪 Sexual Coercion | 0.29^***^ | 0.06 | [0.18, 0.41] |
| Antisocial Behavior 🡪 Frequency of ASF | -0.00 | 0.04 | [-0.09, 0.08] |
| Hypersexuality 🡪 Sexual Coercion | 0.14^***^ | 0.04 | [0.07, 0.21] |
| Hypersexuality 🡪 Frequency of ASF | 0.23^***^ | 0.03 | [0.17, 0.28] |
| Frequency of ASF 🡪 Sexual Coercion | 0.21^***^ | 0.03 | [0.16, 0.27] |
|  | *r* | *SE* | 95% CI |
| Sexual Abuse ↔ Physical Abuse | 0.33^***^ | 0.04 | [0.25, 0.41] |
| Hypersexuality ↔ Antisocial Behavior | 0.35^***^ | 0.04 | [0.28, 0.42] |

*Note.* ^***^*p*<.001; ^**^*p*<.01; ^*^*p*<.05

Table S10

*Model 3: Extended Model Using the Frequency of ASF as Manifest Variable. Standardized Estimates.*

| Factors | β | *SE* | 95% CI |
| --- | --- | --- | --- |
| Physical Abuse 🡪 Antisocial Behavior | 0.28^***^ | 0.03 | [0.22, 0.33] |
| Physical Abuse 🡪 Callous-Unemotional Traits | 0.18^***^ | 0.03 | [0.12, 0.24] |
| Sexual Abuse 🡪 Hypersexuality | 0.01 | 0.03 | [-0.04, 0.07] |
| Callous-Unemotional Traits 🡪 Antisocial Behavior | 0.62^***^ | 0.03 | [0.57, 0.67] |
| Callous-Unemotional Traits 🡪 Rape-Supportive Attitudes | 0.28^***^ | 0.03 | [0.22, 0.33] |
| Callous-Unemotional Traits 🡪 Frequency of ASF | 0.03 | 0.03 | [-0.04, 0.09] |
| Callous-Unemotional Traits 🡪 Violent Pornography Consumption | 0.14^***^ | 0.04 | [0.07, 0.22] |
| Callous-Unemotional Traits 🡪 Hypersexuality | 0.20^***^ | 0.03 | [0.14, 0.26] |
| Antisocial Behavior 🡪 Sexual Coercion | 0.36^***^ | 0.03 | [0.30, 0.43] |
| Antisocial Behavior 🡪 Distorted Perceptions | 0.16^***^ | 0.03 | [0.11, 0.21] |
| Antisocial Behavior 🡪 Frequency of ASF | 0.05 | 0.03 | [-0.02, 011] |
| Antisocial Behavior 🡪 Violent Pornography Consumption | -0.03 | 0.04 | [-0.10, 0.05] |
| Hypersexuality 🡪 Distorted Perceptions | 0.03 | 0.03 | [-0.02, 0.09] |
| Hypersexuality 🡪 Frequency of ASF | 0.11^***^ | 0.03 | [0.06, 0.16] |
| Hypersexuality 🡪 Violent Pornography Consumption | 0.21^***^ | 0.03 | [0.16, 0.26] |
| Distorted Perceptions 🡪 Sexual Coercion | -0.01 | 0.03 | [-0.06, 0.05] |
| Rape-Supportive Attitudes 🡪 Sexual Coercion | 0.19^***^ | 0.04 | [0.12, 0.27] |
| Frequency of ASF 🡪 Sexual Coercion | 0.16^***^ | 0.03 | [0.10, 0.22] |
| Violent Pornography Consumption 🡪 Frequency of ASF | 0.29^***^ | 0.02 | [0.25, 0.33] |
|  | *r* | *SE* | 95% CI |
| Sexual Abuse ↔ Physical Abuse | 0.32^***^ | 0.04 | [0.24, 0.40] |
| Hypersexuality ↔ Antisocial Behavior | 0.35^***^ | 0.04 | [0.27, 0.42] |
| Frequency of Coercive Fantasies ↔ Distorted Perceptions | 0.25^***^ | 0.02 | [0.21, 0.30] |
| Rape-Supportive Attitudes ↔ Frequency of Coercive Fantasies | 0.35^***^ | 0.02 | [0.30, 0.40] |
| Rape-Supportive Attitudes ↔ Distorted Perceptions | 0.31^***^ | 0.03 | [0.26, 0.36] |

*Note.* ^***^*p*<.001; ^**^*p*<.01; ^*^*p*<.05

Table S11

*Model 4: Extended Model with All Pathways Allowed and Estimated Using the Frequency of ASF as Manifest Variable. Standardized Estimates.*

| Factors | β | *SE* | 95% CI |
| --- | --- | --- | --- |
| Physical Abuse 🡪 Antisocial Behavior | 0.30^***^ | 0.03 | [0.24, 0.36] |
| Physical Abuse 🡪 Callous-Unemotional Traits | 0.18^***^ | 0.03 | [0.11, 0.24] |
| Physical Abuse 🡪 Distorted Perceptions | 0.06 | 0.03 | [-0.01, 0.12] |
| Physical Abuse 🡪 Sexual Coercion | 0.07 | 0.05 | [-0.02, 0.16] |
| Physical Abuse 🡪 Rape-Supportive Attitudes | 0.02 | 0.03 | [-0.05, 0.09] |
| Physical Abuse 🡪 Frequency of ASF | 0.03 | 0.03 | [-0.02, 0.08] |
| Physical Abuse 🡪 Violent Pornography Consumption | 0.02 | 0.03 | [-0.04, 0.07] |
| Physical Abuse 🡪 Hypersexuality | 0.05 | 0.03 | [-0.02, 0.11] |
| Sexual Abuse 🡪 Antisocial Behavior | -0.05 | 0.03 | [-0.10, 0.01] |
| Sexual Abuse 🡪 Callous-Unemotional Traits | -0.03 | 0.03 | [-0.09, 0.03] |
| Sexual Abuse 🡪 Distorted Perceptions | 0.02 | 0.03 | [-0.03, 0.07] |
| Sexual Abuse 🡪 Sexual Coercion | 0.11^*^ | 0.05 | [0.01, 0.22] |
| Sexual Abuse 🡪 Rape-Supportive Attitudes | 0.09^*^ | 0.04 | [0.02, 0.16] |
| Sexual Abuse 🡪 Frequency of ASF | 0.04 | 0.02 | [-0.01, 0.08] |
| Sexual Abuse 🡪 Violent Pornography Consumption | 0.03 | 0.02 | [-0.02, 0.07] |
| Sexual Abuse 🡪 Hypersexuality | -0.01 | 0.03 | [-0.08, 0.05] |
| Callous-Unemotional Traits 🡪 Antisocial Behavior | 0.59^***^ | 0.03 | [0.54, 0.64] |
| Callous-Unemotional Traits 🡪 Distorted Perceptions | 0.14^***^ | 0.04 | [0.06, 0.21] |
| Callous/Unemotional Traits 🡪 Sexual Coercion | -0.03 | 0.05 | [-0.13, 0.08] |
| Callous-Unemotional Traits 🡪 Rape-Supportive Attitudes | 0.17^***^ | 0.04 | [0.09, 0.26] |
| Callous-Unemotional Traits 🡪 Frequency of ASF | 0.04 | 0.03 | [-0.03, 0.10] |
| Callous-Unemotional Traits 🡪 Violent Pornography Consumption | 0.11^**^ | 0.04 | [0.04, 0.18] |
| Callous-Unemotional Traits 🡪 Hypersexuality | 0.15^***^ | 0.03 | [0.09, 0.22] |
| Antisocial Behavior 🡪 Sexual Coercion | 0.30^***^ | 0.06 | [0.18, 0.41] |
| Antisocial Behavior 🡪 Distorted Perceptions | 0.01 | 0.04 | [-0.08, 0.09] |
| Antisocial Behavior 🡪 Rape-Supportive Attitudes | 0.02 | 0.05 | [-0.08, 0.12] |
| Antisocial Behavior 🡪 Frequency of ASF | 0.01 | 0.04 | [-0.07, 0.08] |
| Antisocial Behavior 🡪 Violent Pornography Consumption | -0.03 | 0.04 | [-0.11, 0.06] |
| Hypersexuality 🡪 Distorted Perceptions | 0.06^*^ | 0.03 | [0.01, 0.12] |
| Hypersexuality 🡪 Sexual Coercion | 0.12^**^ | 0.04 | [0.05, 0.19] |
| Hypersexuality 🡪 Rape-Supportive Attitudes | 0.15^***^ | 0.03 | [0.09, 0.21] |
| Hypersexuality 🡪 Frequency of ASF | 0.15^***^ | 0.03 | [0.10, 0.20] |
| Hypersexuality 🡪 Violent Pornography Consumption | 0.22^***^ | 0.03 | [0.17, 0.27] |
| Violent Pornography Consumption 🡪 Distorted Perceptions | 0.16^***^ | 0.02 | [0.12, 0.21] |
| Violent Pornography Consumption 🡪 Rape-Supportive Attitudes | 0.24^***^ | 0.02 | [0.19, 0.29] |
| Violent Pornography Consumption 🡪 Sexual Coercion | -0.00 | 0.03 | [-0.06, 0.05] |
| Violent Pornography Consumption 🡪 Frequency of ASF | 0.36^***^ | 0.02 | [0.32, 0.40] |
| Distorted Perceptions 🡪 Sexual Coercion | 0.01 | 0.03 | [-0.05, 0.06] |
| Rape-Supportive Attitudes 🡪 Sexual Coercion | 0.17^***^ | 0.04 | [0.09, 0.25] |
| Frequency of ASF 🡪 Sexual Coercion | 0.15^***^ | 0.03 | [0.09, 0.21] |
|  | *r* | *SE* | 95% CI |
| Sexual Abuse ↔ Physical Abuse | 0.33^***^ | 0.04 | [0.25, 0.42] |
| Hypersexuality ↔ Antisocial Behavior | 0.35^***^ | 0.04 | [0.28, 0.42] |
| Frequency of ASF ↔ Distorted Perceptions | 0.24^***^ | 0.02 | [0.20, 0.28] |
| Rape-Supportive Attitudes ↔ Frequency of Coercive Fantasies | 0.32^***^ | 0.02 | [0.27, 0.37] |
| Rape-Supportive Attitudes ↔ Distorted Perceptions | 0.27^***^ | 0.03 | [0.21, 0.32] |

*Note.* ^***^*p*<.001; ^**^*p*<.01; ^*^*p*<.05

Table S12

*Model 1: Replication of the Original Model Using the Evaluation of ASF as Manifest Indicator. Standardized Estimates.*

| Factors | β | *SE* | 95% CI |
| --- | --- | --- | --- |
| Physical and Emotional Abuse 🡪 Antisocial Behavior | 0.28^***^ | 0.03 | [0.22, 0.34] |
| Physical and Emotional Abuse 🡪 Callous-Unemotional Traits | 0.18^***^ | 0.03 | [0.12, 0.23] |
| Sexual Abuse 🡪 Hypersexuality | 0.01 | 0.03 | [-0.04, 0.07] |
| Callous-Unemotional Traits 🡪 Antisocial Behavior | 0.62^***^ | 0.03 | [0.57, 0.67] |
| Callous-Unemotional Traits 🡪 Evaluation of ASF | 0.17^***^ | 0.04 | [0.10, 0.24] |
| Callous-Unemotional Traits 🡪 Hypersexuality | 0.25^***^ | 0.04 | [0.18, 0.33] |
| Antisocial Behavior 🡪 Sexual Coercion | 0.39^***^ | 0.03 | [0.33, 0.45] |
| Antisocial Behavior 🡪 Evaluation of ASF | 0.01 | 0.04 | [-0.06, 0.08] |
| Hypersexuality 🡪 Evaluation of Coercion | 0.20^***^ | 0.03 | [0.15, 0.25] |
| Evaluation of ASF 🡪 Sexual Coercion | 0.24^***^ | 0.03 | [0.19, 0.30] |
|  | *r* | *SE* | 95% CI |
| Sexual Abuse ↔ Physical Abuse | 0.32^***^ | 0.04 | [0.24, 0.40] |

*Note.* ^***^*p*<.001; ^**^*p*<.01; ^*^*p*<.05

Table S13

*Model 2: Model with All Pathways Allowed and Estimated Using the Evaluation of ASF as Manifest Indicator. Standardized Estimates.*

| Factors | β | *SE* | 95% CI |
| --- | --- | --- | --- |
| Physical Abuse 🡪 Antisocial Behavior | 0.30^***^ | 0.03 | [0.23, 0.36] |
| Physical Abuse 🡪 Callous-Unemotional Traits | 0.18^***^ | 0.03 | [0.11, 0.24] |
| Physical Abuse 🡪 Sexual Coercion | 0.07 | 0.05 | [-0.02, 0.16] |
| Physical Abuse 🡪 Evaluation of ASF | 0.06 | 0.03 | [-0.01, 0.12] |
| Physical Abuse 🡪 Hypersexuality | 0.05 | 0.03 | [-0.02, 0.11] |
| Sexual Abuse 🡪 Antisocial Behavior | -0.05 | 0.03 | [-0.10, 0.00] |
| Sexual Abuse 🡪 Callous-Unemotional Traits | -0.03 | 0.03 | [-0.09, 0.03] |
| Sexual Abuse 🡪 Sexual Coercion | 0.12^*^ | 0.06 | [0.02, 0.23] |
| Sexual Abuse 🡪 Evaluation of ASF | 0.08^**^ | 0.03 | [0.03, 0.14] |
| Sexual Abuse 🡪 Hypersexuality | -0.01 | 0.03 | [-0.08, 0.05] |
| Callous-Unemotional Traits 🡪 Antisocial Behavior | 0.58^***^ | 0.03 | [0.54, 0.63] |
| Callous/Unemotional Traits 🡪 Sexual Coercion | -0.01 | 0.06 | [-0.12, 0.10] |
| Callous-Unemotional Traits 🡪 Evaluation of ASF | 0.22^***^ | 0.04 | [0.15, 0.29] |
| Callous-Unemotional Traits 🡪 Hypersexuality | 0.16^***^ | 0.03 | [0.10, 0.22] |
| Antisocial Behavior 🡪 Sexual Coercion | 0.30^***^ | 0.06 | [0.19, 0.42] |
| Antisocial Behavior 🡪 Evaluation of Coercion | -0.08 | 0.04 | [-0.16, 0.01] |
| Hypersexuality 🡪 Sexual Coercion | 0.15^***^ | 0.04 | [0.08, 0.22] |
| Hypersexuality 🡪 Evaluation of ASF | 0.23^***^ | 0.03 | [0.18, 0.29] |
| Evaluation of ASF 🡪 Sexual Coercion | 0.21^***^ | 0.03 | [0.15, 0.26] |
|  | *r* | *SE* | 95% CI |
| Sexual Abuse ↔ Physical Abuse | 0.33^***^ | 0.04 | [0.25, 0.42] |
| Hypersexuality ↔ Antisocial Behavior | 0.35^***^ | 0.04 | [0.28, 0.42] |

*Note.* ^***^*p*<.001; ^**^*p*<.01; ^*^*p*<.05

Table S14

*Model 3: Extended Model Using the Evaluation of the ASF as Manifest Indicator. Standardized Estimates.*

| Factors | β | *SE* | 95% CI |
| --- | --- | --- | --- |
| Physical Abuse 🡪 Antisocial Behavior | 0.27^***^ | 0.03 | [0.21, 0.33] |
| Physical Abuse 🡪 Callous-Unemotional Traits | 0.18^***^ | 0.03 | [0.12, 0.24] |
| Sexual Abuse 🡪 Hypersexuality | 0.02 | 0.03 | [-0.04, 0.07] |
| Callous-Unemotional Traits 🡪 Antisocial Behavior | 0.61^***^ | 0.03 | [0.56, 0.66] |
| Callous-Unemotional Traits 🡪 Rape-Supportive Attitudes | 0.27^***^ | 0.03 | [0.21, 0.33] |
| Callous-Unemotional Traits 🡪 Evaluation of ASF | 0.18^***^ | 0.03 | [0.11, 0.24] |
| Callous-Unemotional Traits 🡪 Violent Pornography Consumption | 0.14^***^ | 0.04 | [0.07, 0.22] |
| Callous-Unemotional Traits 🡪 Hypersexuality | 0.20^***^ | 0.03 | [0.14, 0.26] |
| Antisocial Behavior 🡪 Sexual Coercion | 0.37^***^ | 0.03 | [0.31, 0.43] |
| Antisocial Behavior 🡪 Distorted Perceptions | 0.16^***^ | 0.03 | [0.11, 0.21] |
| Antisocial Behavior 🡪 Evaluation of Coercion | -0.01 | 0.03 | [-0.08, 0.05] |
| Antisocial Behavior 🡪 Violent Pornography Consumption | -0.02 | 0.04 | [-0.10, 0.05] |
| Hypersexuality 🡪 Distorted Perceptions | 0.03 | 0.03 | [-0.02, 0.09] |
| Hypersexuality 🡪 Evaluation of ASF | 0.10^***^ | 0.03 | [0.05, 0.15] |
| Hypersexuality 🡪 Violent Pornography Consumption | 0.20^***^ | 0.03 | [0.15, 0.25] |
| Distorted Perceptions 🡪 Sexual Coercion | -0.00 | 0.03 | [-0.06, 0.06] |
| Rape-Supportive Attitudes 🡪 Sexual Coercion | 0.18^***^ | 0.04 | [0.10, 0.26] |
| Evaluation of ASF 🡪 Sexual Coercion | 0.14^***^ | 0.04 | [0.07, 0.21] |
| Violent Pornography Consumption 🡪 Evaluation of ASF | 0.22^***^ | 0.02 | [0.18, 0.26] |
|  | *r* | *SE* | 95% CI |
| Sexual Abuse ↔ Physical Abuse | 0.32^***^ | 0.04 | [0.24, 0.40] |
| Hypersexuality ↔ Antisocial Behavior | 0.35^***^ | 0.04 | [0.28, 0.42] |
| Evaluation of Coercion ↔ Distorted Perceptions | 0.32^***^ | 0.02 | [0.28, 0.36] |
| Rape-Supportive Attitudes ↔ Evaluation of Coercion | 0.46^***^ | 0.02 | [0.41, 0.50] |
| Rape-Supportive Attitudes ↔ Distorted Perceptions | 0.30^***^ | 0.03 | [0.25, 0.36] |

*Note.* ^***^*p*<.001; ^**^*p*<.01; ^*^*p*<.05

Table S15

*Model 4: Extended Model with All Pathways Allowed and Estimated Using the Evaluation of ASF as Manifest Indicator. Standardized Estimates.*

| Factors | β | *SE* | 95% CI |
| --- | --- | --- | --- |
| Physical Abuse 🡪 Antisocial Behavior | 0.30^***^ | 0.03 | [0.24, 0.36] |
| Physical Abuse 🡪 Callous-Unemotional Traits | 0.18^***^ | 0.03 | [0.11, 0.24] |
| Physical Abuse 🡪 Distorted Perceptions | 0.06 | 0.03 | [-0.01, 0.12] |
| Physical Abuse 🡪 Sexual Coercion | 0.07 | 0.05 | [-0.03, 0.16] |
| Physical Abuse 🡪 Rape-Supportive Attitudes | 0.02 | 0.03 | [-0.04, 0.08] |
| Physical Abuse 🡪 Evaluation of ASF | 0.05 | 0.03 | [-0.01, 0.10] |
| Physical Abuse 🡪 Violent Pornography Consumption | 0.02 | 0.03 | [-0.04, 0.07] |
| Physical Abuse 🡪 Hypersexuality | 0.05 | 0.03 | [-0.02, 0.11] |
| Sexual Abuse 🡪 Antisocial Behavior | -0.05 | 0.03 | [-0.10, 0.00] |
| Sexual Abuse 🡪 Callous-Unemotional Traits | -0.03 | 0.03 | [-0.09, 0.03] |
| Sexual Abuse 🡪 Distorted Perceptions | 0.02 | 0.03 | [-0.03, 0.07] |
| Sexual Abuse 🡪 Sexual Coercion | 0.12^*^ | 0.05 | [0.01, 0.22] |
| Sexual Abuse 🡪 Rape-Supportive Attitudes | 0.09^**^ | 0.04 | [0.02, 0.16] |
| Sexual Abuse 🡪 Evaluation of ASF | 0.08^**^ | 0.02 | [0.03, 0.12] |
| Sexual Abuse 🡪 Violent Pornography Consumption | 0.03 | 0.02 | [-0.02, 0.08] |
| Sexual Abuse 🡪 Hypersexuality | -0.01 | 0.03 | [-0.08, 0.05] |
| Callous-Unemotional Traits 🡪 Antisocial Behavior | 0.59^***^ | 0.03 | [0.54, 0.64] |
| Callous-Unemotional Traits 🡪 Distorted Perceptions | 0.14^***^ | 0.04 | [0.06, 0.21] |
| Callous/Unemotional Traits 🡪 Sexual Coercion | -0.04 | 0.06 | [-0.14, 0.07] |
| Callous-Unemotional Traits 🡪 Rape-Supportive Attitudes | 0.17^***^ | 0.04 | [0.09, 0.26] |
| Callous-Unemotional Traits 🡪 Evaluation of ASF | 0.19^***^ | 0.03 | [0.12, 0.25] |
| Callous-Unemotional Traits 🡪 Violent Pornography Consumption | 0.11^**^ | 0.04 | [0.04, 0.18] |
| Callous-Unemotional Traits 🡪 Hypersexuality | 0.16^***^ | 0.03 | [0.09, 0.22] |
| Antisocial Behavior 🡪 Sexual Coercion | 0.31^***^ | 0.06 | [0.19, 0.43] |
| Antisocial Behavior 🡪 Distorted Perceptions | 0.01 | 0.04 | [-0.08, 0.09] |
| Antisocial Behavior 🡪 Rape-Supportive Attitudes | 0.01 | 0.05 | [-0.09, 0.11] |
| Antisocial Behavior 🡪 Evaluation of ASF | -0.07 | 0.04 | [-0.15, 0.01] |
| Antisocial Behavior 🡪 Violent Pornography Consumption | -0.03 | 0.04 | [-0.11, 0.06] |
| Hypersexuality 🡪 Distorted Perceptions | 0.06^*^ | 0.03 | [0.01, 0.12] |
| Hypersexuality 🡪 Sexual Coercion | 0.13^**^ | 0.04 | [0.05, 0.20] |
| Hypersexuality 🡪 Rape-Supportive Attitudes | 0.15^***^ | 0.03 | [0.09, 0.21] |
| Hypersexuality 🡪 Evaluation of ASF | 0.17^***^ | 0.03 | [0.12, 0.22] |
| Hypersexuality 🡪 Violent Pornography Consumption | 0.21^***^ | 0.03 | [0.16, 0.27] |
| Violent Pornography Consumption 🡪 Distorted Perceptions | 0.16^***^ | 0.02 | [0.12, 0.21] |
| Violent Pornography Consumption 🡪 Rape-Supportive Attitudes | 0.24^***^ | 0.02 | [0.19, 0.28] |
| Violent Pornography Consumption 🡪 Sexual Coercion | 0.02 | 0.03 | [-0.04, 0.07] |
| Violent Pornography Consumption 🡪 Evaluation of ASF | 0.32^***^ | 0.02 | [0.28, 0.36] |
| Distorted Perceptions 🡪 Sexual Coercion | 0.01 | 0.03 | [-0.05, 0.07] |
| Rape-Supportive Attitudes 🡪 Sexual Coercion | 0.16^***^ | 0.04 | [0.07, 0.24] |
| Evaluation of ASF 🡪 Sexual Coercion | 0.12^**^ | 0.04 | [0.05, 0.19] |
|  | *r* | *SE* | 95% CI |
| Sexual Abuse ↔ Physical Abuse | 0.33^***^ | 0.04 | [0.25, 0.42] |
| Hypersexuality ↔ Antisocial Behavior | 0.35^***^ | 0.04 | [0.28, 0.42] |
| Evaluation of Coercion ↔ Distorted Perceptions | 0.29^***^ | 0.02 | [0.25, 0.34] |
| Rape-Supportive Attitudes ↔ Evaluation of ASF | 0.43^***^ | 0.02 | [0.38, 0.47] |
| Rape-Supportive Attitudes ↔ Distorted Perceptions | 0.26^***^ | 0.03 | [0.21, 0.31] |

*Note.* Indirect effects are shown in Table S5 below. ^***^*p*<.001; ^**^*p*<.01; ^*^*p*<.05

Table S16

*Model 1: Replication of the Original Model Based on a Random Sample with One Participant from Each Family (N=2,513). Standardized Estimates.*

| Factors | β | *SE* | 95% CI |
| --- | --- | --- | --- |
| Physical and Emotional Abuse 🡪 Antisocial Behavior | 0.31^***^ | 0.03 | [0.24, 0.37] |
| Physical and Emotional Abuse 🡪 Callous-Unemotional Traits | 0.17^***^ | 0.03 | [0.11, 0.24] |
| Sexual Abuse 🡪 Hypersexuality | 0.04 | 0.03 | [-0.02, 0.10] |
| Callous-Unemotional Traits 🡪 Antisocial Behavior | 0.61^***^ | 0.03 | [0.55, 0.67] |
| Callous-Unemotional Traits 🡪 Aggressive Sexual Fantasies | 0.10^*^ | 0.05 | [0.00, 0.20] |
| Callous-Unemotional Traits 🡪 Hypersexuality | 0.28^***^ | 0.05 | [0.19, 0.38] |
| Antisocial Behavior 🡪 Sexual Coercion | 0.39^***^ | 0.04 | [0.32, 0.46] |
| Antisocial Behavior 🡪 Aggressive Sexual Fantasies | 0.09 | 0.05 | [-0.00, 0.18] |
| Hypersexuality 🡪 Aggressive Sexual Fantasies | 0.29^***^ | 0.04 | [0.22, 0.36] |
| Aggressive Sexual Fantasies 🡪 Sexual Coercion | 0.28^***^ | 0.04 | [0.20, 0.35] |
|  | *r* | *SE* | 95% CI |
| Sexual Abuse ↔ Physical Abuse | 0.33^***^ | 0.05 | [0.24, 0.42] |

*Note..*^***^*p*<.001; ^**^*p*<.01; ^*^*p*<.05

Table S17

*Model 2: Model with All Pathways Allowed and Estimated Based on a Random Sample with One Participant from Each Family (N=2,513). Standardized Estimates.*

| Factors | β | *SE* | 95% CI |
| --- | --- | --- | --- |
| Physical Abuse 🡪 Antisocial Behavior | 0.33^***^ | 0.04 | [0.25, 0.40] |
| Physical Abuse 🡪 Callous-Unemotional Traits | 0.17^***^ | 0.04 | [0.10, 0.24] |
| Physical Abuse 🡪 Sexual Coercion | 0.07 | 0.05 | [-0.04, 0.17] |
| Physical Abuse 🡪 Aggressive Sexual Fantasies | 0.03 | 0.04 | [-0.06, 0.11] |
| Physical Abuse 🡪 Hypersexuality | 0.08^*^ | 0.04 | [0.01, 0.16] |
| Sexual Abuse 🡪 Antisocial Behavior | -0.05 | 0.03 | [-0.11, 0.01] |
| Sexual Abuse 🡪 Callous-Unemotional Traits | -0.02 | 0.03 | [-0.08, 0.04] |
| Sexual Abuse 🡪 Sexual Coercion | 0.14^*^ | 0.06 | [0.01, 0.26] |
| Sexual Abuse 🡪 Aggressive Sexual Fantasies | 0.10^**^ | 0.04 | [0.03, 0.18] |
| Sexual Abuse 🡪 Hypersexuality | -0.01 | 0.04 | [-0.08, 0.06] |
| Callous-Unemotional Traits 🡪 Antisocial Behavior | 0.57^***^ | 0.03 | [0.51, 0.62] |
| Callous/Unemotional Traits 🡪 Sexual Coercion | -0.00 | 0.06 | [-0.11, 0.11] |
| Callous-Unemotional Traits 🡪 Aggressive Sexual Fantasies | 0.17^***^ | 0.05 | [0.08, 0.26] |
| Callous-Unemotional Traits 🡪 Hypersexuality | 0.19^***^ | 0.04 | [0.12, 0.26] |
| Antisocial Behavior 🡪 Sexual Coercion | 0.32^***^ | 0.07 | [0.19, 0.45] |
| Antisocial Behavior 🡪 Aggressive Sexual Fantasies | -0.01 | 0.06 | [-0.11, 0.10] |
| Hypersexuality 🡪 Sexual Coercion | 0.08 | 0.04 | [-0.01, 0.17] |
| Hypersexuality 🡪 Aggressive Sexual Fantasies | 0.28^***^ | 0.04 | [0.21, 0.36] |
| Aggressive Sexual Fantasies 🡪 Sexual Coercion | 0.23^***^ | 0.04 | [0.15, 0.32] |
|  | *r* | *SE* | 95% CI |
| Sexual Abuse ↔ Physical Abuse | 0.34^***^ | 0.05 | [0.24, 0.43] |
| Hypersexuality ↔ Antisocial Behavior | 0.35^***^ | 0.04 | [0.28, 0.43] |

*Note.* ^***^*p*<.001; ^**^*p*<.01; ^*^*p*<.05

Table S18

*Model 3: Extended Model Based on a Random Sample with One Participant from Each Family (N=2,513). Standardized Estimates.*

| Factors | β | *SE* | 95% CI |
| --- | --- | --- | --- |
| Physical Abuse 🡪 Antisocial Behavior | 0.30^***^ | 0.03 | [0.23, 0.36] |
| Physical Abuse 🡪 Callous-Unemotional Traits | 0.18^***^ | 0.03 | [0.12, 0.24] |
| Sexual Abuse 🡪 Hypersexuality | 0.03 | 0.03 | [-0.03, 0.09] |
| Callous-Unemotional Traits 🡪 Antisocial Behavior | 0.60^***^ | 0.03 | [0.55, 0.66] |
| Callous-Unemotional Traits 🡪 Rape-Supportive Attitudes | 0.26^***^ | 0.03 | [0.19, 0.32] |
| Callous-Unemotional Traits 🡪 Aggressive Sexual Fantasies | 0.13^***^ | 0.04 | [0.05, 0.21] |
| Callous-Unemotional Traits 🡪 Violent Pornography Consumption | 0.11^*^ | 0.04 | [0.02, 0.19] |
| Callous-Unemotional Traits 🡪 Hypersexuality | 0.24^***^ | 0.04 | [0.17, 0.31] |
| Antisocial Behavior 🡪 Sexual Coercion | 0.39^***^ | 0.04 | [0.32, 0.46] |
| Antisocial Behavior 🡪 Distorted Perceptions | 0.17^***^ | 0.03 | [0.11, 0.23] |
| Antisocial Behavior 🡪 Aggressive Sexual Fantasies | 0.04 | 0.04 | [-0.04, 013] |
| Antisocial Behavior 🡪 Violent Pornography Consumption | 0.01 | 0.04 | [-0.07, 0.10] |
| Hypersexuality 🡪 Distorted Perceptions | 0.03 | 0.03 | [-0.03, 0.10] |
| Hypersexuality 🡪 Aggressive Sexual Fantasies | 0.12^**^ | 0.04 | [0.05, 0.19] |
| Hypersexuality 🡪 Violent Pornography Consumption | 0.20^***^ | 0.03 | [0.14, 0.26] |
| Distorted Perceptions 🡪 Sexual Coercion | -0.05 | 0.04 | [-0.12, 0.02] |
| Rape-Supportive Attitudes 🡪 Sexual Coercion | 0.11^*^ | 0.06 | [0.00, 0.22] |
| Aggressive Sexual Fantasies 🡪 Sexual Coercion | 0.20^**^ | 0.06 | [0.09, 0.31] |
| Violent Pornography Consumption 🡪 Aggressive Sexual Fantasies | 0.38^***^ | 0.03 | [0.32, 0.43] |
|  | *r* | *SE* | 95% CI |
| Sexual Abuse ↔ Physical Abuse | 0.33^***^ | 0.05 | [0.24, 0.42] |
| Hypersexuality ↔ Antisocial Behavior | 0.35^***^ | 0.04 | [0.27, 0.43] |
| Aggressive Sexual Fantasies ↔ Distorted Perceptions | 0.45^***^ | 0.03 | [0.39, 0.50] |
| Rape-Supportive Attitudes ↔ Aggressive Sexual Fantasies | 0.61^***^ | 0.03 | [0.55, 0.68] |
| Rape-Supportive Attitudes ↔ Distorted Perceptions | 0.30^***^ | 0.03 | [0.24, 0.36] |

*Note.* ^***^*p*<.001; ^**^*p*<.01; ^*^*p*<.05

Table S19

*Model 4: Extended Model with All Pathways Allowed and Estimated Based on a Random Sample with One Participant from Each Family (N=2,513). Standardized Estimates.*

| Factors | β | *SE* | 95% CI |
| --- | --- | --- | --- |
| Physical Abuse 🡪 Antisocial Behavior | 0.33^***^ | 0.04 | [0.26, 0.40] |
| Physical Abuse 🡪 Callous-Unemotional Traits | 0.17^***^ | 0.04 | [0.10, 0.24] |
| Physical Abuse 🡪 Distorted Perceptions | 0.05 | 0.04 | [-0.03, 0.13] |
| Physical Abuse 🡪 Sexual Coercion | 0.06 | 0.05 | [-0.04, 0.17] |
| Physical Abuse 🡪 Rape-Supportive Attitudes | 0.02 | 0.04 | [-0.06, 0.10] |
| Physical Abuse 🡪 Aggressive Sexual Fantasies | 0.02 | 0.04 | [-0.05, 0.09] |
| Physical Abuse 🡪 Violent Pornography Consumption | 0.00 | 0.04 | [-0.07, 0.07] |
| Physical Abuse 🡪 Hypersexuality | 0.08* | 0.04 | [0.01, 0.16] |
| Sexual Abuse 🡪 Antisocial Behavior | -0.05 | 0.03 | [-0.10, 0.01] |
| Sexual Abuse 🡪 Callous-Unemotional Traits | -0.02 | 0.03 | [-0.08, 0.04] |
| Sexual Abuse 🡪 Distorted Perceptions | 0.03 | 0.03 | [-0.03, 0.10] |
| Sexual Abuse 🡪 Sexual Coercion | 0.13^*^ | 0.06 | [0.01, 0.26] |
| Sexual Abuse 🡪 Rape-Supportive Attitudes | 0.09^*^ | 0.04 | [0.01, 0.18] |
| Sexual Abuse 🡪 Aggressive Sexual Fantasies | 0.09^**^ | 0.03 | [0.03, 0.16] |
| Sexual Abuse 🡪 Violent Pornography Consumption | 0.03 | 0.03 | [-0.03, 0.08] |
| Sexual Abuse 🡪 Hypersexuality | -0.01 | 0.04 | [-0.07, 0.06] |
| Callous-Unemotional Traits 🡪 Antisocial Behavior | 0.57^***^ | 0.03 | [0.52, 0.63] |
| Callous-Unemotional Traits 🡪 Distorted Perceptions | 0.12^***^ | 0.04 | [0.05, 0.20] |
| Callous/Unemotional Traits 🡪 Sexual Coercion | -0.01 | 0.06 | [-0.12, 0.10] |
| Callous-Unemotional Traits 🡪 Rape-Supportive Attitudes | 0.15^**^ | 0.04 | [0.06, 0.24] |
| Callous-Unemotional Traits 🡪 Aggressive Sexual Fantasies | 0.13^**^ | 0.04 | [0.05, 0.21] |
| Callous-Unemotional Traits 🡪 Violent Pornography Consumption | 0.08^*^ | 0.04 | [0.00, 0.15] |
| Callous-Unemotional Traits 🡪 Hypersexuality | 0.19^***^ | 0.04 | [0.11, 0.26] |
| Antisocial Behavior 🡪 Sexual Coercion | 0.32^***^ | 0.07 | [0.19, 0.46] |
| Antisocial Behavior 🡪 Distorted Perceptions | 0.03 | 0.05 | [-0.07, 0.13] |
| Antisocial Behavior 🡪 Rape-Supportive Attitudes | 0.01 | 0.06 | [-0.10, 0.13] |
| Antisocial Behavior 🡪 Aggressive Sexual Fantasies | -0.01 | 0.05 | [-0.11, 0.09] |
| Antisocial Behavior 🡪 Violent Pornography Consumption | 0.02 | 0.05 | [-0.07, 0.11] |
| Hypersexuality 🡪 Distorted Perceptions | 0.05 | 0.03 | [-0.01, 0.11] |
| Hypersexuality 🡪 Sexual Coercion | 0.07 | 0.04 | [-0.01, 0.16] |
| Hypersexuality 🡪 Rape-Supportive Attitudes | 0.16^***^ | 0.04 | [0.09, 0.23] |
| Hypersexuality 🡪 Aggressive Sexual Fantasies | 0.19^***^ | 0.04 | [0.12, 0.26] |
| Hypersexuality 🡪 Violent Pornography Consumption | 0.21^***^ | 0.03 | [0.15, 0.27] |
| Violent Pornography Consumption 🡪 Distorted Perceptions | 0.17^***^ | 0.02 | [0.12, 0.22] |
| Violent Pornography Consumption 🡪 Rape-Supportive Attitudes | 0.23^***^ | 0.03 | [0.18, 0.28] |
| Violent Pornography Consumption 🡪 Sexual Coercion | -0.04 | 0.04 | [-0.12, 0.04] |
| Violent Pornography Consumption 🡪 Aggressive Sexual Fantasies | 0.48^***^ | 0.03 | [0.43, 0.54] |
| Distorted Perceptions 🡪 Sexual Coercion | -0.04 | 0.04 | [-0.11, 0.02] |
| Rape-Supportive Attitudes 🡪 Sexual Coercion | 0.08 | 0.06 | [-0.03, 0.20] |
| Aggressive Sexual Fantasies 🡪 Sexual Coercion | 0.23^***^ | 0.07 | [0.08, 0.37] |
|  | *r* | *SE* | 95% CI |
| Sexual Abuse ↔ Physical Abuse | 0.34^***^ | 0.05 | [0.24, 0.43] |
| Hypersexuality ↔ Antisocial Behavior | 0.35^***^ | 0.04 | [0.28, 0.43] |
| Aggressive Sexual Fantasies ↔ Distorted Perceptions | 0.42^***^ | 0.03 | [0.37, 0.48] |
| Rape-Supportive Attitudes ↔ Aggressive Sexual Fantasies | 0.58^***^ | 0.03 | [0.52, 0.65] |
| Rape-Supportive Attitudes ↔ Distorted Perceptions | 0.26^***^ | 0.03 | [0.20, 0.32] |

*Note.* ^***^*p*<.001; ^**^*p*<.01; ^*^*p*<.05
